# Exploring the Structure of Spatial Representations

**DOI:** 10.1371/journal.pone.0157343

**Published:** 2016-06-27

**Authors:** Tamas Madl, Stan Franklin, Ke Chen, Robert Trappl, Daniela Montaldi

**Affiliations:** 1 School of Computer Science, University of Manchester, Manchester, United Kingdom; 2 Austrian Research Institute for Artificial Intelligence, Vienna, Austria; 3 Institute for Intelligent Systems, University of Memphis, Memphis, United States of America; 4 School of Psychological Sciences, University of Manchester, Manchester, United Kingdom; Chinese Academy of Sciences, CHINA

## Abstract

It has been suggested that the map-like representations that support human spatial memory are fragmented into sub-maps with local reference frames, rather than being unitary and global. However, the principles underlying the structure of these ‘cognitive maps’ are not well understood. We propose that the structure of the representations of navigation space arises from clustering within individual psychological spaces, i.e. from a process that groups together objects that are close in these spaces. Building on the ideas of representational geometry and similarity-based representations in cognitive science, we formulate methods for learning dissimilarity functions (metrics) characterizing participants’ psychological spaces. We show that these learned metrics, together with a probabilistic model of clustering based on the Bayesian cognition paradigm, allow prediction of participants’ cognitive map structures in advance. Apart from insights into spatial representation learning in human cognition, these methods could facilitate novel computational tools capable of using human-like spatial concepts. We also compare several features influencing spatial memory structure, including spatial distance, visual similarity and functional similarity, and report strong correlations between these dimensions and the grouping probability in participants’ spatial representations, providing further support for clustering in spatial memory.

## Introduction

### Motivation

There has been considerable research on spatial representations facilitating navigation since Tolman coined the term ‘cognitive map’ [1]. Since then, the neural bases of such allocentric (world-centered) representations of space have been identified in rats [2, 3] and humans [4, 5] and have been shown to play a vital role in representing locations within the environment in long-term memory. Instead of learning a single spatial map with a global reference frame, as proposed originally [1, 2], humans (as well as some non-human animals) seem to form structured spatial maps, consisting of multiple ‘sub-maps’, i.e. multiple representations containing spatial information about sub-sets of objects in the environment.

Behavioural evidence has suggested that human spatial maps are structured, and has been interpreted as comprising multi-level hierarchies [6–10], or at least as having multiple local reference frames [11, 12]. These hierarchies, extracted from recall sequences, can be observed even in the case of randomly distributed objects with no boundaries [8], with participants’ response times and accuracies being affected by this structure (subjects overestimated distances between objects on different branches of the hierarchy and underestimated distances within branches, and showed shorter response times for within-branch judgements). Further evidence for the existence of multiple representations in different spatial reference frames [11–13] has been derived from the accuracies of judgements of relative direction, which are heavily affected by subjects’ frames of reference.

Furthermore, there is also strong neuroscientific evidence for hierarchical spatial representations [14, 15], and for fragmentation into sub-maps [16] in mammalian brains. Finally, organized and structured maps (instead of a single representation) are consistent with ‘chunking’ in long-term memory [17] and with hierarchical models of cognition [18], and have multiple information processing advantages, including the increased speed and efficiency of retrieval search, and economical storage.

Cognitive map structure is not introspectively accessible nor immediately apparent, and has to be inferred indirectly. Nevertheless, these structures play an important role in spatial cognition. It has been shown in experiments involving priming, distance and angle estimations, and sketch maps, that the speed and accuracy of subjects at various spatially relevant tasks are significantly influenced by how they represent space [6, 8, 19, 20].

In addition to helping us understand these influences on cognitive performance, a model of cognitive map structure could be of interest to several neighbouring fields, including human-robot interaction (allowing robots to use human-like spatial concepts), artificial intelligence (using insights from human memory to improve artificial memory), geographic information science (presenting spatial information in a more easily comprehensible and memorable fashion), or personal navigation aids (allowing specification of goals as human spatial concepts instead of specific addresses, e.g. ‘navigate to the area with the student bars’).

### Structured cognitive maps

In our usage of the terms in this paper, ‘spatial memory’ and ‘cognitive maps’ contain spatial representations of objects in navigation space which are allocentric (world-centered instead of stored in relation to the organism), goal-independent, and are stored long-term. They afford flexible route planning (e.g., detours or shortcuts) and judgements of distances and directions of represented objects. We will use the term ‘sub-map’ to mean one part of such ‘cognitive maps’ (containing a subset of the objects stored in spatial memory that belong together, and their spatial locations), and we use ‘map structure’ to refer to information regarding which objects belong together (on the same sub-map). It has been argued that objects which belong together in spatial memory are also consistently recalled together [6–8], based on prior uses of the free recall paradigm to study memory organization [21, 22]. Thus, in this paper, we determine objects belonging together by extracting those that are always recalled together (see Materials and Methods). Other empirical methods for investigating cognitive map structure are described in the Discussion.

Despite the importance of the question for several fields of cognitive science, it is not well understood how cognitive maps might be structured in non-trivial, open environments. Several types of abstract structure have been proposed, including graphs [23–25], hierarchies [6–10, 26, 27], (semi-)lattices [28, 29], multiple local reference frames [11, 12, 30], or fragments or sub-maps [16, 31].

Some computational models of cognitive map structure have been proposed. Most of them have not been quantitatively evaluated against human data. Examples of abstract formal models include the graph-based model by [32] for outdoor virtual reality environments, or based on image schemas [33] or predicate logic [34] for indoor environments (neither of these have been evaluated against human data). More neuronally plausible but functionally simpler models of structured cognitive maps include the topological graph-based model of place cells by [25] and the hierarchy-based model by [26, 27]. These models have not been compared to empirical data. In contrast, the neural model by [35] can account for lesion effects in humans, but not for large-scale cognitive map structure.

[36] has published the modelling work closest in spirit to the predictive models reported below, utilizing self-organizing maps to model hierarchical cognitive map structure, and reporting that on average, the model exhibits similar distance estimation error patterns to the estimation biases (averaged over all subjects) reported by [6]. However, this model has not been compared to individual subject maps; and is unable to account for per-subject data, since it uses only Euclidean spatial distance and no other features (spatial distance seems to be insufficient to explain individual representation structures).

No consensus exists as to a formal structure best explaining the data. However, there is some common ground. Instead of proposing a single, monolithic ‘cognitive map’, most studies cited above claim that spatial memory contains several sets of objects belonging together in some sense, i.e., on the same sub-map, fragment, branch of a hierarchy, etc. In addition to neural firing patterns changing significantly when transitioning from the vicinity of one such sub-map to another [16, 31], it seems that objects on the same sub-maps are consistently recalled together, even in not explicitly compartmentalized environments [6–8]. This observation makes it possible to make explicit the sub-map structure of individual participants, by asking them to produce recall lists, and looking for sets of objects always recalled together [6] (a methodology based on utilizing item ordering in the free recall paradigm to study memory organization [21, 22]).

Although objects belonging to the same sub-map seem to be located in close vicinity, spatial proximity alone does not suffice to explain which objects belong to which representation in the memories of individuals. The lack of either such an explanation or a predictive model in the existing literature strongly suggests that other features may also play a role. In addition to spatial proximity being insufficient to explain sub-map structure on an individual level, associative learning mechanisms would also suggest other features to influence which objects may be associated in memory. For example, functionally related buildings that are frequently thought of together, such as places to eat close to one’s workplace, may become strongly associated through Hebbian learning. Spatial memories are more useful when connected to non-spatial information about objects, and can indeed be influenced and cued by non-spatial stimuli.

Multiple features influencing map structure have been suggested, including boundaries in the environment [5, 37], spatial distance and familiarity [6], action-based and perception-based similarity [20, 38], and functional / semantic similarity [9]. However, to the authors’ best knowledge, these influences have never been compared based on behavioural data. Furthermore, despite of the above-mentioned evidence for structured cognitive maps, no empirically tested, formally defined model exists that would be able to predict the structure of spatial sub-maps constructed by individual humans in unconstrained large-scale environments. The present paper aims to advance the literature on spatial memory structure by providing the first such comparison of features, and a first predictive computational model.

### Modelling cognitive map structure

Formulating models and testable hypotheses precisely and unambiguously is important for efficiently driving research, especially in interdisciplinary areas such as spatial memory (which is of interest in psychology, neuroscience, and artificial intelligence, among other fields). Computational cognitive models are well suited to this challenge—being unambiguous formal descriptions—and provide a common language across disciplines, as well as the additional advantage of very fast prediction generation and hypothesis testing (once the data has been collected, such models can be rapidly run and verified on computers). Thus, they play an important role in the cognitive science of spatial memory, helping to integrate findings, to generate, define, formalize and test hypotheses, and to guide research [39].

In order to develop and validate a computational model of cognitive map structure, it is necessary to tackle the methodological difficulties associated with indirectly inferring consciously inaccessible spatial representation structure from noisy data. In addition, there are also computational challenges. Just like brains can be said to create object representations based on perceived and remembered properties of objects, a computational cognitive model also needs such representations, capturing relevant features. The structure of the representations in the brain and those in a good model should be similar; that is, objects belonging to the same representation in the brain of a participant should also belong together in the model.

It has been argued that representational geometries can capture both the content and format of representations in brains as well as computational models [40]. The representational geometry of a brain region representing a number of possible objects can be characterised by means of the dissimilarities of the brain-activity patterns corresponding to those objects. This characterisation extends a long history of mathematical and cognitive psychology investigating human representational geometry—also referred to as ‘psychological spaces’—using a notion of [dis]similarity based on behavioural data [41–43] (we use the terms representational geometry and psychological space interchangeably, and use them to mean a metric space in which the dissimilarities of objects can be represented as distances between points). Representational similarity analysis (RSA) [44], a popular framework for quantitatively comparing representations between behaviour, brain imaging, and computational models, is also based on this idea. RSA has recently been applied to the hippocampus (a major brain region associated with spatial memories), showing strong evidence of hierarchical organization of long-term memories [45].

**Fig 1:**
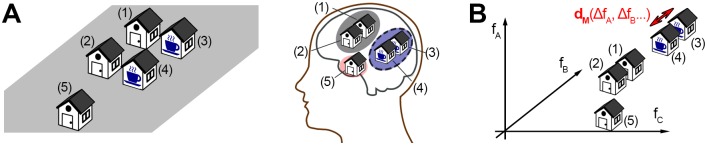
Modelling spatial representation structure using representational geometry. A: Example grouping where a non-spatial feature (functional similarity) plays an important role. Coffee shops (3 and 4) and houses (1 and 2) are grouped together in the respective representations in the illustrated person’s memory (grey/solid and blue/dashed ellipses), due to their shared purpose and close proximity. This representation structure influences spatial cognitive processes, such as distance estimations, planning times, or recall order. B: Representational geometry model. Several features (*f*_*A*_, *f*_*B*_, …), spatial and non-spatial, can influence the organization of spatial memories. An appropriate metric or distance function *d*_*M*_ can quantify the dissimilarity between each pair of buildings, and characterize a representational geometry model, which then represents the buildings as points in a metric space. Spatial representation structure can be inferred by performing clustering (grouping objects in close proximity) within this model. The metric, and thus the psychological space, may differ across individuals.

Adopting the framework of representational geometries, a model of cognitive map structure requires a **dissimilarity function or ‘metric’** characterising the psychological space containing objects stored in allocentric long-term spatial memory (see Fig 1). A metric (or dissimilarity function, or distance function) is a function that defines a non-negative ‘distance’ between pairs of objects, and characterises a metric space (two well-known examples for metrics include the Euclidean distance, which characterises Euclidean space, and the taxicab or Manhattan distance defining *l*_1_ space). As opposed to using the simple Euclidean norm, a more general metric can also account for different importances (or weights) of the features, such as the stronger weighting of functional similarity compared to spatial distance in Fig 1. In addition, a **‘clustering’ mechanism** is needed for grouping together objects which are similar in this space, according to this metric. Without such a mechanism, the model would not able to structure cognitive maps, that is, to assign a subset of objects to the same representation or sub-map in spatial memory.

A computational model of cognitive map structure constituted by a representational geometry model similar to that of a human participant, together with a cognitively plausible grouping mechanism, should be able to not only fit the data of that participant, but to make advance predictions (i.e., to predict which objects will belong together in her cognitive map, based on their features). However, despite of the popularity of RSA and of the idea of structured cognitive maps, neither an empirically validated spatial memory ‘metric’, nor a predictive model of the grouping mechanism underlying spatial memory structure have been published to date.

This paper describes such a model of the structure of cognitive maps, as well as behavioural experiments for investigating the features influencing these structures and for validating the model. We hypothesize that the mechanism responsible for structuring cognitive maps can be modelled by a clustering algorithm (**clustering hypothesis**), and that individual participants may differ in their representational geometry within which this clustering takes place (**individual psychological space hypothesis**).

Our main contributions include:

- Evidence that the spatial memory structures inferred by the recall order paradigm (described below) play a significant role in multiple spatial cognitive processes, including planning, distance estimation, memory accuracy, and response times;
- Comparison of several information types (features) significantly influencing cognitive map structure;
- A metric learning method to acquire the dissimilarity functions characterising the psychological spaces of individual participants, from small amounts of training data obtainable from recall lists;
- The (to our best knowledge) first computational model of cognitive map structure able to predict individual map structures in navigation space, based on the individual psychological space and clustering hypotheses, and supporting evidence. The ability of this model to predict a large majority of subject cognitive map structures substantiates these two hypotheses.

## Materials and Methods

### Experimental paradigm

**Fig 2:**
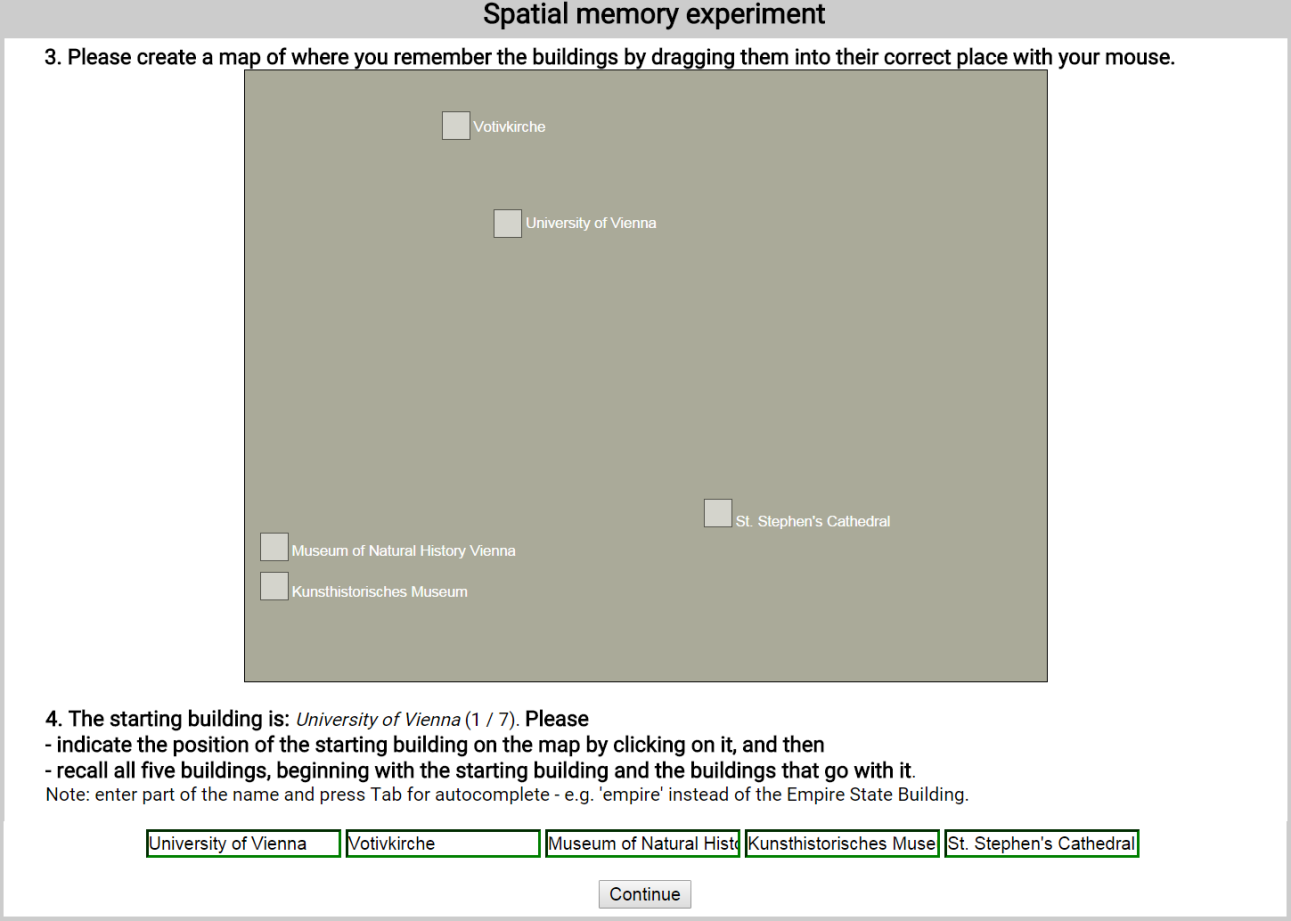
A part of the real-world memories experiment interface of Experiments 1 and 3, with the sketch map question for verifying that subjects have indeed formed allocentric cognitive maps (top), and the recall sequence question requiring them to recall every single building name multiple times (bottom). During this recall question the labelled sketch map was not visible to subjects.

**Fig 3:**
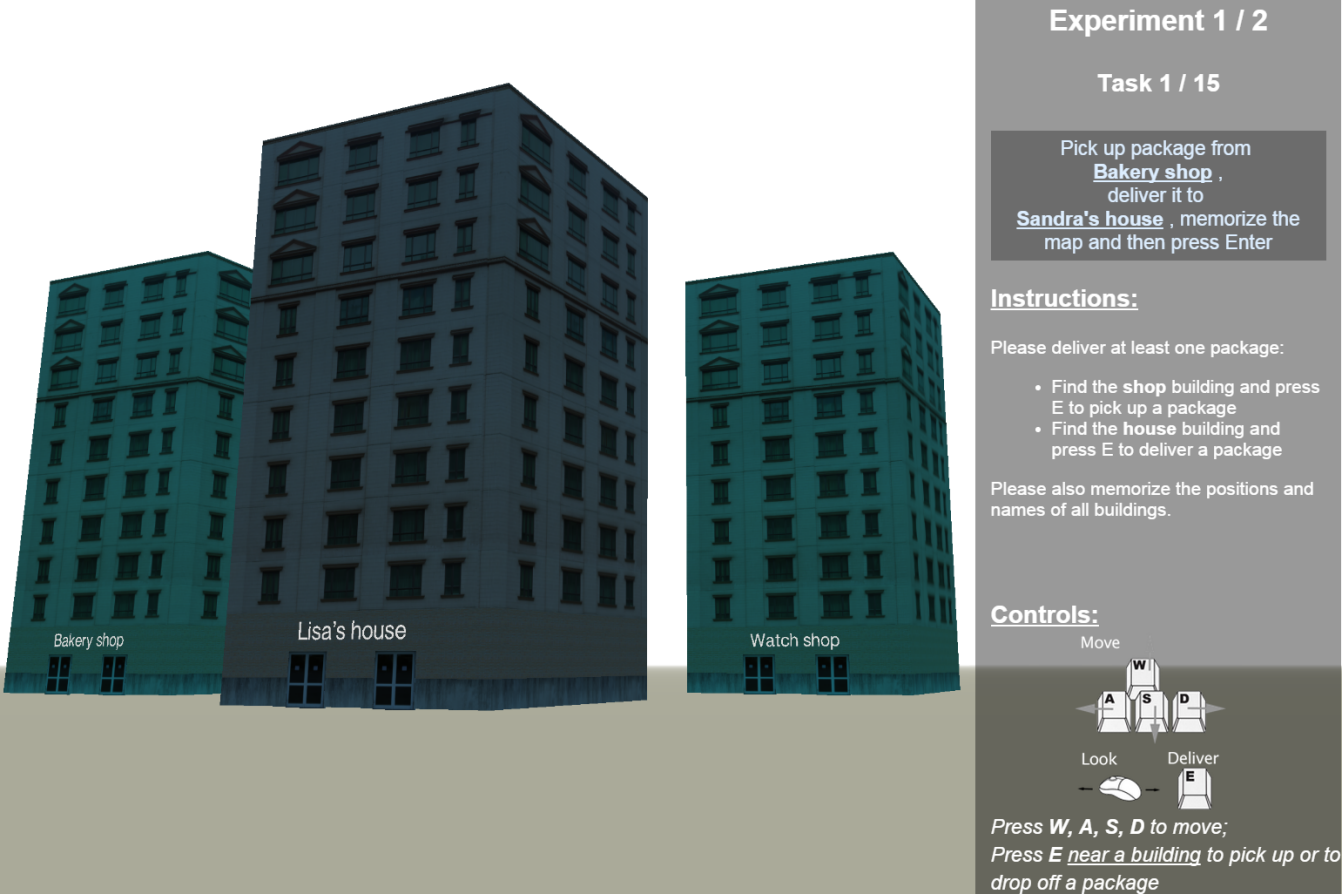
A part of the virtual reality experiment interface of Experiment 2 (the recall sequence interface was equivalent to the real-world experiments; see Fig 2).

We investigated the structure of spatial representations in navigation space in three experiments. All of the experiments were concerned with the representations of buildings and their relation to each other. In Experiments 1 and 3, subjects recalled real-world buildings that they were already highly familiar with (see Fig 2). In Experiment 2, subjects were presented with three-dimensional virtual reality environments—containing buildings with automatically generated properties—which they had to memorize prior to the recall task from which the representation structure was inferred (see Fig 3).

The experiments reported below were approved by the Computer Science Ethics Review Committee of the University of Manchester (application numbers CS163, CS155, and CS173 for Experiments 1, 2 and 3). Participants provided written informed consent for Experiment 2, and confirmed their informed consent in the other experiments in their browsers, through the online experiment platform Amazon Mechanical Turk. The Computer Science Ethics Review Committee approved of this procedure.

### Extraction of spatial representation structure

**Fig 4:**
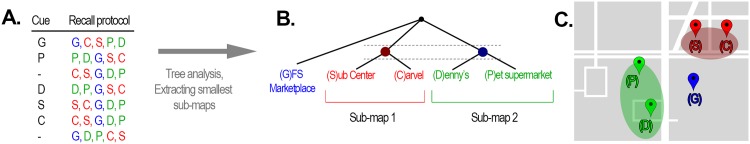
The recall sequence-based method used to extract cognitive map structure. A: Example recall sequences of one of the participants of Experiment 3. Each building was cued once, with two uncued recall trials interspersed (full building names abbreviated by their first character). B: Hierarchical tree structures were constructed by tree analysis, based on the assumption that buildings always recalled together belong to the same sub-map. C: Geographic map of the buildings recalled by this participant. Sub-maps shown in colour, according to the extracted structure.

To extract the structure of spatial representations, we use a variant of ordered tree analysis on subjects’ recall sequences, a behavioural methodology used by [6–9] among others for extracting hierarchies in spatial representations, and by [21, 46] for verbal stimuli. The core assumption behind this methodology is that objects recalled together belong to the same representation; i.e. that on the whole, subjects recall every object within a representation (or sub-map) before moving on to the next representation (see Fig 4). Tree analysis operates on a set of recall sequences (with each sequence consisting of all object names, recalled with a particular ordering—usually different from the other recall sequences -, as exemplified in Fig 2 top). Variety among these recall sequences is encouraged by cueing subjects with the object they are required to start with (and only uncued parts of the sequence are analysed to avoid the interference of the cue) [6].

To briefly summarize the collection of these recall sequences (for details, see Experiment 1): in each trial, subjects were first asked to pick a few buildings (five / eight in Exp. 3B) within walking distance of each other, which they were very familiar with, and such that they knew how to walk from any one building to any other. Subsequently, they were asked to recall the complete list (i.e. recall sequence) of their chosen buildings, starting with a cue building (except for two interspersed uncued trials), multiple times. If building names were missing or incorrect, subjects were prompted again, until they got all of them right. Thus, the ordering within the individual sequences was their only variable aspect.

After obtaining the recall sequences, for each subject, the tree analysis algorithm simply iterates through all possible combinations of subsets of object names in each recall sequence, finds those subsets which consistently appear together in all sequences (regardless of order), and constructs a hierarchy based on containment relationships from the subsets of items occurring together. The original algorithm also extracts directionality information for each group (whether the items within that group have always been recalled using a consistent ordering). We do not use the directionality information in the recall sequences in this work (see S1 File for the algorithm we have used). Fig 4 A shows example abbreviated recall sequences, and the resulting tree structure, where each branch or sub-map consists of items which always occur together in the sequences. Unambiguous sub-map memberships are obtained at the level just above the leaf nodes, defining sub-maps as elementary sets of co-occurring items, i.e. those which do not themselves contain further co-occurring items. This procedure partitions buildings into one or two sub-maps in Experiments 1, 2 and 3A, and up to four sub-maps in Experiment 3B.

Since this tree analysis algorithm requires buildings to be recalled together in every single recall sequence in order to infer subjects’ sub-maps, it is very sensitive to individual inconsistencies that may result from lapses of attention, task interruptions, and other kinds of noise within participant response (see Discussion for an overview and comparison with other approaches of inferring cognitive map structure). To mitigate this, we have eliminated ‘outlier’ recall sequences, defined as sequences which would have statistically significantly altered the structure if they were included (whereas all others would not).

As proposed in previous work on hierarchical cognitive maps [6–8], we used jackknifing to eliminate outliers. For each sequence, this procedure calculates how the inferred tree structure would change if the sequence were omitted. More specifically, from the recall sequences $\left(S_{1},...,S_{N}\right)$ produced by a subject for a specific environment, tree analysis was applied and the tree obtained for the omission of each recall sequence, $\left(T_{1},...,T_{N}\right)$ (where each $T_{i}$ denotes the tree calculated from all recall sequences except $S_{i}$. See S1 File for the tree analysis algorithm). All trees should be the same if there were no outliers.

As suggested by [8], trees were quantified using two statistics, tree height h(T) (the maximum distance from the root) and log-cardinality $c\left(T\right)=\log_{2}\left(\prod_{i=1}^{K}n_{i}!\right)$ (the logarithm of the number of possible recall sequences consistent with that tree), where *n*_*i*_ denotes the number of branches in node *i*, and k is the number of nodes. These statistics were calculated for all trees $\left(T_{1},...,T_{N}\right)$ that would result from possible sequence omissions (i.e. if only sequences excluding the omitted one had been entered by the participant). If any of the sequence omissions lead to a statistically significant change in the tree statistics, at a significance level of *α* = 0.05, then that sequence was deemed an outlier and was omitted, and the tree resulting from the other sequences of that trial was used for further analysis. That is, sequence *i* was excluded if either the z-score of corresponding tree heights $\left(h\left(T_{i}\right)-\bar{h}\right)/\sqrt{\left(Var[h]\right)}$, or the z-score of log-cardinalities $\left(c\left(T_{i}\right)-\bar{c}\right)/\sqrt{\left(Var[c]\right)}$ exceeded the threshold corresponding to *α* = 0.05; where **h** and **c** stand for the vector of all tree heights and log-cardinalities of all possible omissions of single recall lists in the same environment, respectively (We used a less conservative significance criterion than prior work due to the simpler structures and smaller numbers of objects used. Using the extremely conservative significance level of *α* = 0.001 used in the prior work cited above would have led to zero outliers being detected—presumably incorrectly, since it is unlikely that not a single participant would have had any interruptions or lapses of attention.)

All sequences except for outliers were consistent with the same tree structure. Outlier sequences, which significantly changed the tree structure, were likely to arise from the above-mentioned sources of noise (lapses of attention, interruptions, etc.). The outlier sequences detected and removed by the jackknifing procedure comprised 8.5% in Experiment 1, 10.0% in Experiment 2 and 9.5% in Experiment 3, corresponding to less than one omission per subject (across the 7 recall sequences produced per subject and trial).

Due to the reliance on significance testing, and to the small numbers of recall lists collected, the described jackknifing procedure is not able to remove every outlier disrupting inferred map structures. Thus, in some environments, participants’ inferred building groupings may be incorrect. We have estimated the probability of unremoved outliers in the data using simulated distractions, i.e. simulated ‘mistakes’ in the orderings of recall lists, investigating which proportion of them can successfully be recognized and eliminated using jackknifing. Briefly, 6.5% of the outliers in the 5-building condition (A), and 2.5% in the 8-building condition (B) in Experiment 3 are estimated to remain undetected by jackknifing. Due to these undetected outliers in the training and testing data, even a ‘perfect’ computational model would not be able to achieve 100% prediction. Based on the undetected 6.5% and 2.5% outliers, the maximum possible prediction rates are 63% in condition A and 78% in condition B of Experiment 3 according to our simulation (see S1 File).

To simplify the analysis, we subsequently extract the elementary sub-maps (those not containing smaller sub-maps) from the constructed tree—this allows us to model sub-maps, as opposed to full hierarchies. These elementary sub-maps must contain at least two buildings. If a sub-map only contains a single object, then this object is excluded from subsequent analysis. The main reason being that our hypothesis implies sub-maps to be clusters or groups of objects; however, there is no way to verify the plausibility of a single-object cluster (as opposed to clusters containing multiple buildings, for which performance consequences such as between/within-cluster distance biases, priming effects etc. can be investigated—see Experiment 1 for evidence). A further reason for the exclusion of single-object sub-maps is that they were likely to actually be parts of bigger sub-maps in subjects’ spatial memories, together with additional buildings not captured due to the necessarily limited number of recalled items per trial in our experiments. The exclusion of these single buildings did not have an impact on the plausibility of our claims, since two sub-maps containing pairs of buildings suffice for comparing within sub-map and across sub-map estimations in order to investigate whether representation structure inferred from recall order has an effect on spatial cognition (see Experiment 1. Exp. 3B collected map structures with eight buildings and up to four sub-maps to show that the model is not limited to two).

A final difference between our methodology and prior uses of the recall order paradigm is the repetition with several different geographic environments for each subject in Experiment 3. Repeatedly extracting cognitive map structures from the same participants is not only interesting, e.g. to compare the variability of the features in subjects’ psychological spaces, but also of vital importance for producing and validating a predictive model of the structure of spatial representations. Given the large inter-subject variability in terms of features and feature importances influencing map structure, parametrizing such a predictive model necessitates gathering multiple different cognitive map structures from separate environments (and not just one structure), both for training the model, and for subsequently testing it. The main differences between a repeated and a single-trial paradigm include possible effects of fatigue due to the increased length of experiments, as well as declining accuracy of representations towards the later stages (participants started struggling to cue readily available buildings which they could accurately draw on a map beyond 20 buildings, as evidenced by much slower progress, higher error rates, and much higher rate of participants abandoning the experiment as compared to Experiment 1 which used single trials).

An attempt to mitigate these effects—as well as practical limitations—motivated the decision to use a smaller number of buildings (five in Experiments 1 and 2, five and eight in 3 A and B) compared to the single-trial setup of [6, 8], who used 32 and 28 objects, respectively. Using their dozens of buildings for each of the five or three map structures of Experiment 3 would have required participants to recall (and accurately localize) around one hundred buildings or more—as well as judging all of their pairwise similarities, the number of which increases quadratically with the number of buildings (in the case of 32 buildings, they would amount to 496 similarity judgements each for visual and functional similarities, and for each trial, which is nowhere near feasible).

### Experimental platforms and participants

Participants in two of the three Experiments (1 and 3) were recruited from the online survey website Amazon Mechanical Turk (MTurk—https://www.mturk.com). Multiple psychological findings have been replicated before, using subjects from MTurk [47], showing the breadth of this platform for psychological experimentation. MTurk offers a participant pool that is significantly more diverse than samples of university students, containing subjects from many countries worldwide and of different age groups; as well being several orders of magnitude larger than most universities’ subject pools. But the most important advantage offered by this platform lay in facilitating the collection of information about spatial representations of many, very different geographic environments. Such variety is critical for two main reasons:

- To facilitate generalizable observations (for example, insights from inflexibly planned city areas such as the grid layout of Manhattan might not have been generalizable to other street layouts), and
- To avoid local biases (for example, using exclusively local maps in the same city for each participant might have led to conclusions about the spatial structure of the local city, reflected in subjects’ representations, as opposed to insights into the way subjects structure space in general).

**Fig 5:**
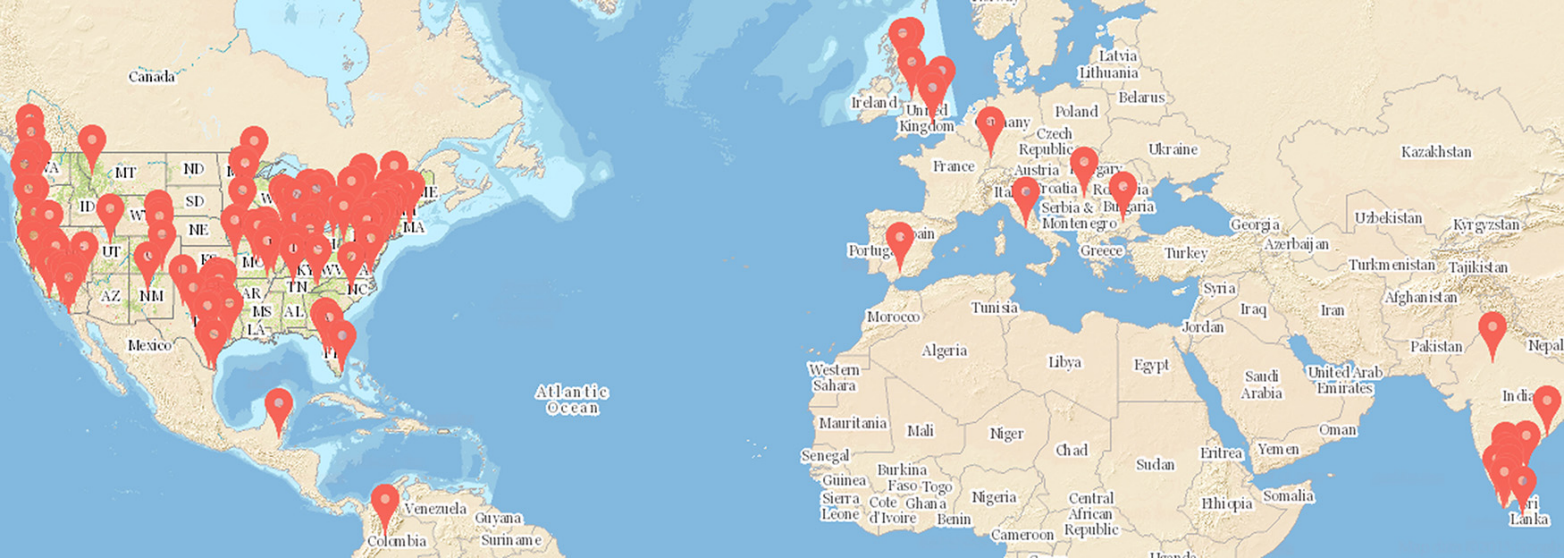
Overview over the 149 cities in which participants’ spatial memory structures were extracted (and predicted by the computational model) in the real-world experiments (full list in S1 File).

Our objective of collecting cognitive map structures from a large variety of different geographical environments was indeed successful—we collected data and analysed spatial representations from several environments within **149 different cities** across multiple continents (see Fig 5—a list of these cities can be found in S1 File).

### Exclusion of participant maps not significantly better than random chance

Throughout this paper, we have only analysed participants’ data if their sketch maps were significantly better than random chance, in order to avoid false conclusions about cognitive maps being made on the basis of non-allocentric representations. Since route knowledge suffices for navigating between buildings, participants might have lacked survey knowledge about some of the buildings in these experiments. To rule out participant data not showing evidence of allocentric cognitive maps, we first performed a test of participants’ sketch maps against randomness, before carrying out the subsequent analyses described in the Results section.

We compared the sum of squared errors (SSE) calculated by subtracting the positions of buildings on participants’ sketch maps from those on the correct geographical map (obtained from Google Maps), with the SSEs of 10,000 randomly generated maps containing 5 buildings against the correct map. Since subjects’ sketch maps were produced on empty surfaces without any position, orientation or scale cues, as seen in Fig 2, they were first aligned (translated, rotated, and scaled) with the correct map using Procrustes analysis [48] without reflection. The randomly generated maps were also aligned in the same fashion. The distribution of the SSEs of 10,000 Procrustes-aligned random maps was then used to test whether subject maps were better than random (further increasing the number of maps, e.g. to 20,000, did not make a difference to the results). Specifically, subject SSEs were tested against the null hypothesis that they were drawn from the distribution of uniformly random map SSEs. Two different significance tests were applied at *α* = 0.05 significance level, and found to largely agree (in all but 3% of the cases in Experiment 1, 1.3% in Exp. 2 and 4% in Exp. 3): a Z-test assuming normal distributions of SSEs, and a non-parametric Bootstrap hypothesis test [49], which requires subject maps to be better than a proportion of 1 − *α* of the random maps.

Because the former test makes the assumption of normally distributed data, which is incorrect for the vast majority of distributions of random map SSEs according to Shapiro-Wilk tests, we use the latter, non-parametric hypothesis testing method throughout this paper to test participant maps against randomness.

### Computational model

As outlined in the introduction, we use a representational geometry model to account for participants’ spatial representation structure. In particular, the model consists of two parts: 1) a dissimilarity function or ‘metric’ which can calculate distances for any two objects, given their features, and allows constructing a ‘psychological space’ within which objects can be represented as points with these distances; and 2) a clustering mechanism for grouping together points in this space. The subsections below describe the employed metrics and clustering algorithms.

Distance metrics defining subjects’ psychological spaces do not constitute mechanistic models of cognition—they do not clearly correspond to neural processing (they operate on Marr’s algorithmic level). Nor are they isomorph to the real world in the manner of image schemas. Rather, they are models seeking second-order isomorphisms [50, 51]. That is, correspondence is sought between the similarities of represented objects in the model and those represented in participants’ memory, not between distal stimuli and their proximal representations. Nevertheless, such models aiming for second-order isomorphisms have been successful at modelling and predicting human behaviour, and have even been argued to be superior to models directly aiming for veridical representations [42].

Two different kinds of dissimilarity functions (metrics) are used below: linear metrics (based on linearly weighted Euclidean distance), and nonlinear metrics (based on a Gaussian model expressing the co-representation probability of pairs of objects). The former is the simplest way to extend the standard Euclidean metric to account for different feature importances, but is limited to representational geometries that can be obtained by affine transforms of the feature space (as they are linear models). Little is known about the representational geometry of navigation-space spatial memory, but there is strong evidence that other neural representations employ nonlinear transformations [52]. For this reason, a nonlinear metric is also evaluated below. Leveraging a Gaussian model for this metric is both mathematically straightforward and plausible for systems adding up large numbers of inputs—which include many models of the brain—due to the Central Limit Theorem (which states that the sum of a large number of independent variables will be approximately Gaussian, regardless of their actual distribution).

#### Passively learning linear metrics characterizing psychological spaces

Automatically learning human representation similarity metrics (or psychological spaces) from highly noisy and sparse data is a largely unexplored problem in the cognitive sciences. Given full similarity matrices obtained from subjects (all possible pairwise similarities), there is a popular approach for projecting data into a space in which distances accurately reflect these similarities, called multidimensional scaling (MDS) [53]. This method is not applicable in our case, because it requires a full pairwise distance matrix, whereas our training data comes from several different environments. Pairwise distances and similarities are only known within, and not across, those environments. Furthermore, it is not straightforward to generalize from known similarities to those of novel objects in an MDS model.

In machine learning and statistics, the Mahalanobis distance is frequently used as a general metric which is able to produce distances (dissimilarities) for novel pairs of objects, and can account for weighted features. Below, we will assume that the features of all objects can be described using vectors $x=(\begin{array}{c} f_{A}\\ f_{B}\\ ... \end{array})$ consisting of feature values *f*_*A*_, *f*_*B*_, …, and will denote the vector of pairwise differences between two objects as $\Delta x=x_{1}-x_{2}=\left(\begin{array}{c} \Delta f_{A}\\ \Delta f_{B}\\ ... \end{array}\right)$. The Mahalanobis distance is defined as

$$\begin{array}{c} d\left(x_{1},x_{2}\right)=d_{M}\left(\Delta x\right)=\sqrt{\Delta x^{T}W\Delta x}, \end{array}$$ (1)

where *W* is the inverse of the covariance matrix expressing the importance of each feature, and their influences on each other. Assuming a diagonal inverse covariance matrix (i.e., independent features), that is, $W=(\begin{array}{ccc} w_{A} & 0 & ...\\ 0 & w_{B} & ...\\ ... & \end{array})$, this expression reduces to a metric that is equivalent to a weighted Euclidean distance:

$$\begin{array}{c} d_{M}\left(\Delta x\right)=\sqrt{w_{A}\Delta f_{A}^{2}+w_{B}\Delta f_{B}^{2}+...}, \end{array}$$ (2)

where the weights *w*_∗_ express the importance of each feature (this expression would equal the usual Euclidean distance if all *w*_∗_ = 1). Once these weights are known, Eq (2) allows calculating the distance for any pair of novel objects (provided the pairwise difference Δ**x** of their features is known). As a metric, it fully characterises a metric space, and suffices to define a psychological space modelling the representational geometry of a human participant—provided the weights actually correspond to the relative importances attributed to these features by that participant.

**Fig 6:**
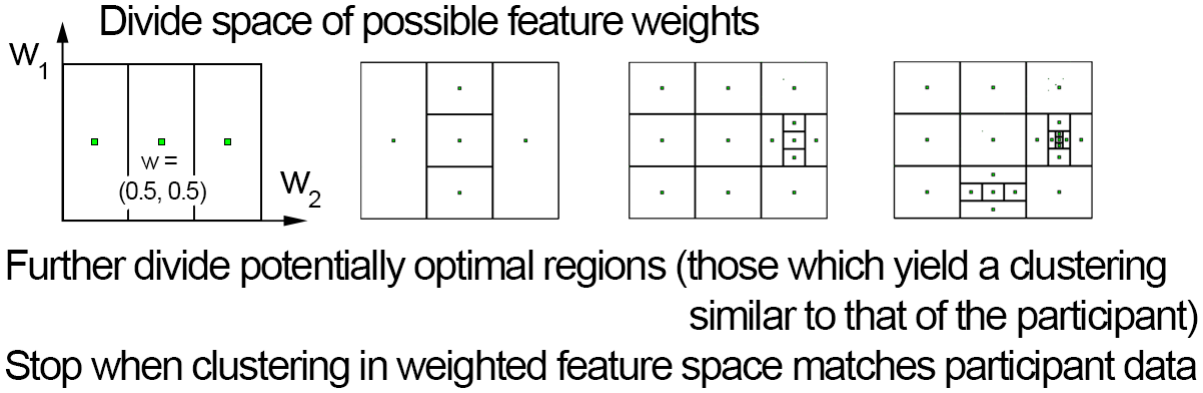
Passively learning a linear metric. Weights for a linear metric can be obtained by searching for the optimal weights using global optimization, e.g. the DIRECT (DIviding RECTangles) [55] method illustrated here. This method keeps dividing the space of possible feature weights into thirds, and further divides potentially optimal regions (those which minimize the objective function—in our case, weights that parametrize a metric which yields a grouping close to participant’s map structure), until the weights best matching participant training data (producing groupings most similar to participants’ actual representation structure) are found.

Thus, the computational problem of acquiring such a metric reduces to learning the weights *w*_*A*_, *w*_*B*_, … from the available, known sub-map memberships of buildings. In particular, the weights have to be adjusted such that any pair of buildings known to be co-represented on the same sub-map yields a smaller distance than any pair of buildings represented on different sub-maps. The easiest way to do so is using an off-the-shelf **global optimization** approach to learn these parameters of a linear metric. We have used the locally biased variant [54] of DIRECT (DIviding RECTangles) [55], a global, deterministic, derivative-free optimization method based on Lipschitzian optimization, which can handle the kinds of non-linear and non-convex functions which clustering accuracy inevitably entails. DIRECT finds global optima by systematically dividing the feature space into smaller and smaller hyperrectangles, returning the one yielding the best results upon convergence (see Fig 6).

**Fig 7:**
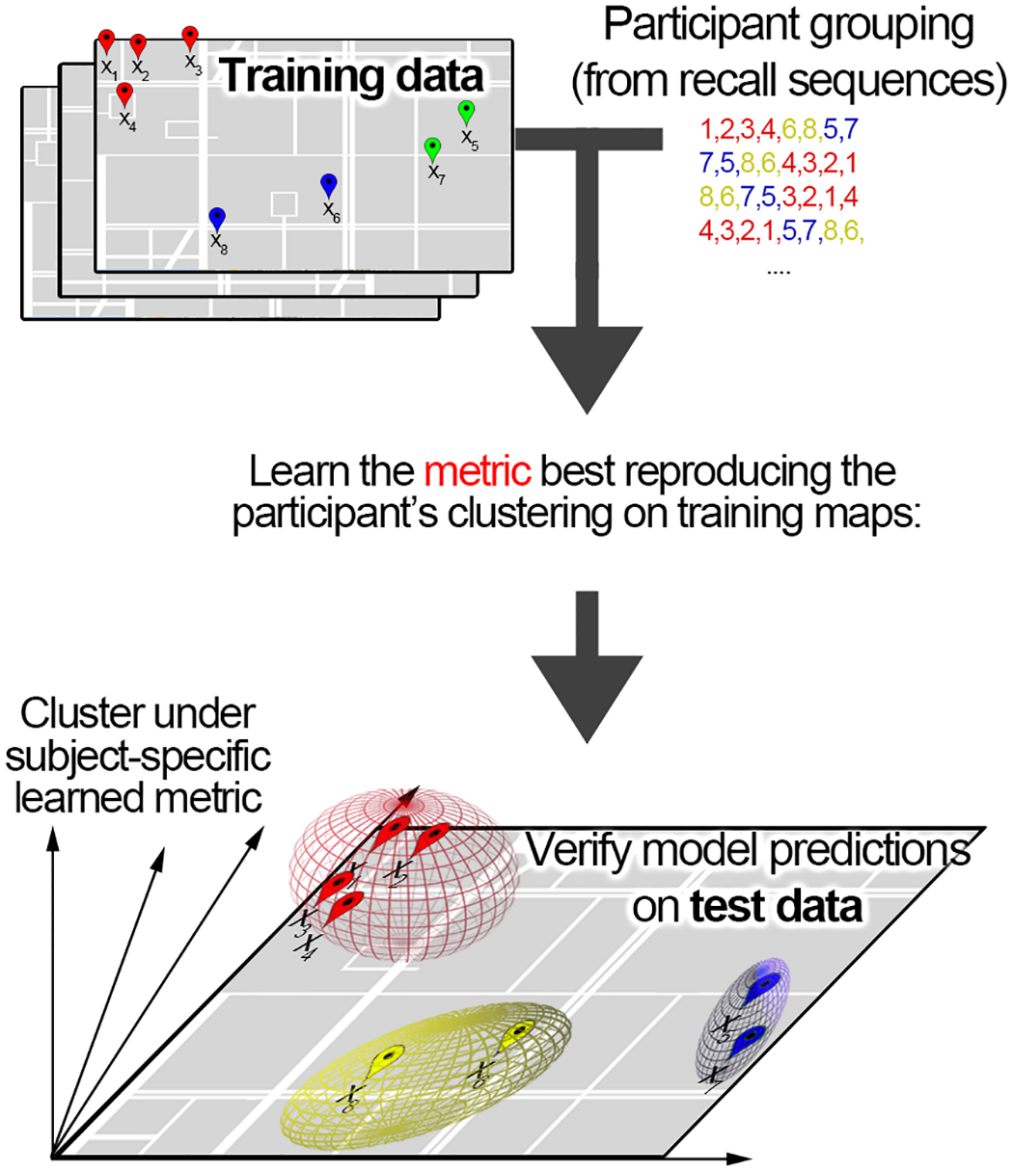
Training and testing procedure for learning a metric and evaluating it against participant data.

To summarize the procedure of passively learning and verifying a linear metric defining a psychological space model (see also Fig 7 for an illustration of the learning / testing procedure):

1. Collect map structures / groupings *M* (information which buildings belonging together in a participants’ memory) from *n* environments, using the ordered tree paradigm.
2. Based on the features of the buildings (see Results), collect all pairwise difference vectors *D* = [Δ**x**_1,2_ Δ**x**_1,3_ …] between all building pairs in each environment.
3. Split the data *M* and *D* from the *n* environments into training data *M*_*train*_ and *D*_*train*_, from which the subject-specific model (metric) will be learned, and test data *M*_*test*_ and *D*_*test*_, which will be used for evaluating the model.
4. Define an objective function *o*(**w**, *M*_*train*_, *D*_*train*_), which, given a set of parameters **w** (in the linear case, feature weights) as well as map structures *M*_*train*_ and differences *D*_*train*_, returns the clustering accuracy (a numerical value expressing how closely a clustering of the buildings under the given metric corresponds to the correct grouping imposed by the participant).
5. Find the weights best modelling the participant data using global optimization: $w_{best}=\underset{\Delta w}{\text{argmin}}\;o\left(w,M,D\right)$.
6. Evaluate the model characterized by the linear metric with parameters **w**_*best*_, by applying clustering under this metric to the test data *D*_*test*_, and comparing the model predictions with the participant groupings *M*_*test*_.

Clustering accuracies are quantified using Rand indices [56]. The Rand index is a measure of the amount of correctly assigned pairs among all pairs, and is defined as $R=\left(s+d\right)/\left(\begin{array}{c} B\\ 2 \end{array}\right)$, where *B* is the total number of buildings on a map structure, *s* is the number of building pairs on the same sub-map both in the predicted and actual map structure, and *d* the pairs on different sub-maps both in prediction and in subject data.

An automatic learning procedure similar to steps 1-6 above was applied in the following two sections, varying only step 5 (the procedure for finding the parameters of the metric). Instead of using global optimization, in the case of active learning, the parameters minimizing the model uncertainty are calculated, and in the case of passive non-linear metrics, a Gaussian model was used to learn dissimilarity functions for each subject (see below) instead of optimizing the weights of a linear metric.

#### Actively learning linear metrics characterizing psychological spaces

In experimental paradigms allowing full control of the environment to be memorized by participants (such as Experiment 2), one way to tackle the challenge of learning accurate metrics from few data points involves generating the environments such that participants’ subsequent responses minimize the uncertainty of the model regarding the feature importances, inspired by active learning in machine learning [57]. The idea is to alternate between acquiring a model from known groupings of buildings by a participant, and generating an environment based on it, such that model uncertainty is reduced as much as possible (because of the optimal reduction of uncertainty, this method can be expected to learn a better model from less data points than global optimization). After subjects have been queried on a reasonable number of generated environments, and the model’s uncertainty regarding their psychological space has decreased, they are presented with completely random environments, on which the trained models are tested.

**Fig 8:**
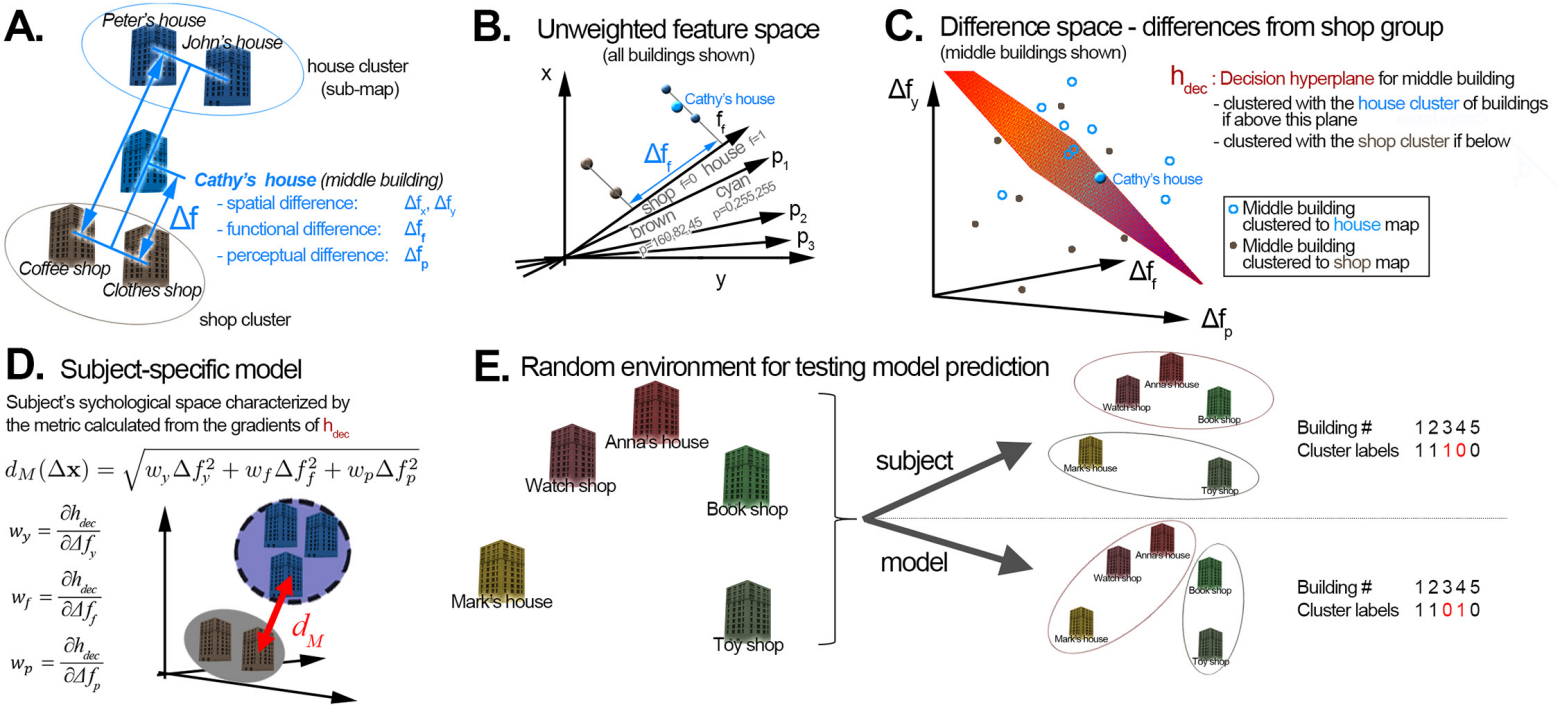
The decision hyperplane method for inferring feature importances and generating environments in Experiment 2. A: General layout of training trials, which consisted of two groups of two buildings (with equal colour and function within a group), and a middle building, the parameters of which could be varied (distance, similarity in colour and in function to the shop group). B: Feature space representation—each building can be represented as a single point **x** in a space spanned by the features (position, colour, function). C: Difference space representation—middle buildings can be represented in terms of their difference to the shop group along each feature. According to the clustering hypothesis, there has to be a ‘decision hyperplane’ calculable from these middle buildings, such that those below the plane (i.e. those closer to the shop group) are most likely clustered with the shop group, and those above the plane (i.e. farther away from the shop group) are most likely clustered with the house group. D. Subject-specific models consist of a metric, in this case a weighted Euclidean distance (with weights expressing the importance of each feature to that subject), and a clustering algorithm. The weights can be calculated from the decision boundary—the importance of each feature is proportional to the derivative (slope) of the decision boundary by that feature. E. Randomly generated testing environments, and comparison procedure. Subjects impose a grouping even on random, unstructured environments, as shown by previous research [8]. Based on this metric, a clustering model produces a grouping. Subsequently, cluster labels are compared (and, in this example, found to be incorrect).

We have implemented an active linear metric learning approach, the **decision hyperplane method**, based on this idea, using generated virtual reality environments in Experiment 2. We constructed a training environment for each trial such that 1) they contained two clusters (shop buildings and house buildings), 2) only the features of a single building, which lay somewhere between the two clusters, were varied (see Fig 8). We trained a linear classifier to assign the middle buildings of all trials of a participant to one or the other cluster in feature space. The class label (dependent variable) *y* was derived from that participant’s recall sequences in each trial (*y* = 1 if the middle building was co-represented—i.e. recalled together—with the shop buildings, and 0 if it was co-represented with the house buildings). The differences between the middle building and the shop buildings along all features (in unweighted feature space) served as predictor (independent) variables Δ**x**.

Based on these variables, a linear ‘decision hyperplane’ was calculated, which separated the set of all data points characterizing the middle buildings of a participant’s trials into two sets: into middle buildings which were represented together with shops (if below the decision hyperplane) and into those which were co-represented with houses (if above the decision hyperplane)—see Fig 8. The slope of this ‘decision hyperplane’ in each feature dimension (distance, visual similarity / colour similarity, functional similarity) thus indicated the importance of each feature to this participant (for example, if the decision hyperplane in Fig 8 was horizontal, that would mean that the y-axis—spatial distance—would be the only feature of relevance for this subject. Conversely, if the plane was almost vertical, spatial distance would be unimportant).

The decision hyperplane was calculated using logistic regression [58], formulating the question whether to group the middle building on the shop sub-map or the house sub-map as a binary classification problem. Thus, the probability *P*(*S* = 1|Δ**x**) of clustering the middle building to the shop sub-map, given the pairwise differences Δ**x** = (Δ*f*_*s*_, Δ*f*_*f*_, Δ*f*_*p*_, …) from the shop buildings along a number of features, including spatial (Δ*f*_*s*_), functional (Δ*f*_*f*_) and perceptual (Δ*f*_*p*_) difference (i.e., difference in colour in Experiment 2), was modelled using the logistic regression equation

$$\begin{array}{c} P\left(S=1|\Delta x\right)=\frac{1}{1+e^{-w^{T}\Delta x}}, \end{array}$$ (3)

in which the model parameters $w=(\begin{array}{c} w_{s}\\ w_{f}\\ w_{p}\\ ... \end{array})$ control the slope of the decision hyperplane, and thus represent participants’ feature importances in this model. After learning the decision hyperplane, these model parameters were used to construct the metric in Eq (2) characterizing the participant’s ‘psychological space’, which leads to attenuated differences along features unimportant to the participant.

The accuracy of the learned weights **w** depends on where known data points (i.e. the pairs of buildings for which it is known whether or not they belong to the same representation) are located in the feature space. The linear model described above assumes that for a building located between two different groups of buildings, there is a specific linear hyperplane in feature space, such that points on one side of the hyperplane belong with the first group and points on the other side belong to the second group (see Fig 8).

In order to obtain the most information from the fewest possible environments, they should be generated such that they minimize the model’s uncertainty regarding feature importances, once the associated grouping in the participant’s psychological space becomes apparent from her response. To achieve this, the parameters of the new middle building were drawn from the region of the currently calculated decision hyperplane, since this is the region in which the model is least certain as to where buildings should be assigned. As the region of least certainty, or greatest uncertainty, comprises the points with a classification probability of 0.5 to either class, these points can be defined as: $\Delta x_{LC}=\underset{\Delta x}{\text{argmin}}\;\left|0.5-P\left(S|\Delta x\right)\right|$. From this and Eq (3), it follows that **w**^*T*^Δ**x**_*LC*_ = 0, i.e. that points of least certainty lie on the hyperplane described by **w**. Formally, this is equivalent to active learning [57] with uncertainty sampling [59] in machine learning.

The experiment actively learning a linear metric (Experiment 2) employ the same training and testing procedure outlined in the previous subsection (see Fig 7), with two differences. First, the environments generated to collect the training data (*M*_*train*_ and *D*_*train*_) are created based on the current decision hyperplane (always taking into account all training data collected so far). In contrast, the environments generated to collect the test data are created with completely random features.

Second, instead of using global optimization and a clustering-based objective function, the weights are calculated using linear regression. The best decision hyperplane (the one most consistent with the data) is learned by maximizing the probability of Eq (3) over all known data points (middle buildings), i.e. $w_{best}=\arg\max_{w}\prod_{i=1}^{N}P{\left(S=1|\Delta x_{i}\right)}^{S_{i}}{\left(1-P\left(S=1|\Delta x_{i}\right)\right)}^{1-S_{i}}$, where *S*_*i*_ is 1 if the i’th middle building is a shop, and 0 otherwise. The best parameters can be calculated using gradient descent [58].

#### Passively learning non-linear metrics characterizing psychological spaces

Because of existing evidence for non-linear transformations employed in the brain’s representational geometry, e.g. in the visual domain [52], it is worthwhile to extend the above modelling procedure to non-linear metrics. Metric learning [60] in machine learning is concerned with finding a distance metric—such as linear, Mahalanobis distance metrics, and their associated parameters (see above), or non-linear metrics by projecting the data into kernel space using e.g. a Radial Basis Function (RBF) kernel Φ in a distance function $d_{RBF}\left(x_{1},x_{2}\right)=\sqrt{{\left(\Phi \left(x_{1}\right)-\Phi \left(x_{2}\right)\right)}^{⊺}\left(\Phi \left(x_{1}\right)-\Phi \left(x_{2}\right)\right)}$, e.g. [61, 62].

Here, we propose a novel metric, for the following reasons. First, the popular RBF-based metric requires variances to be isotropic, i.e. to not differ much across features (since the RBF kernel uses a single parameter instead of a full covariance matrix, it cannot fit non-isotropic data well—see [63]). This assumption does not hold in our data (perceptual similarities usually vary more than functional similarities, and both vary orders of magnitude less than spatial distances between buildings).

**Fig 9:**
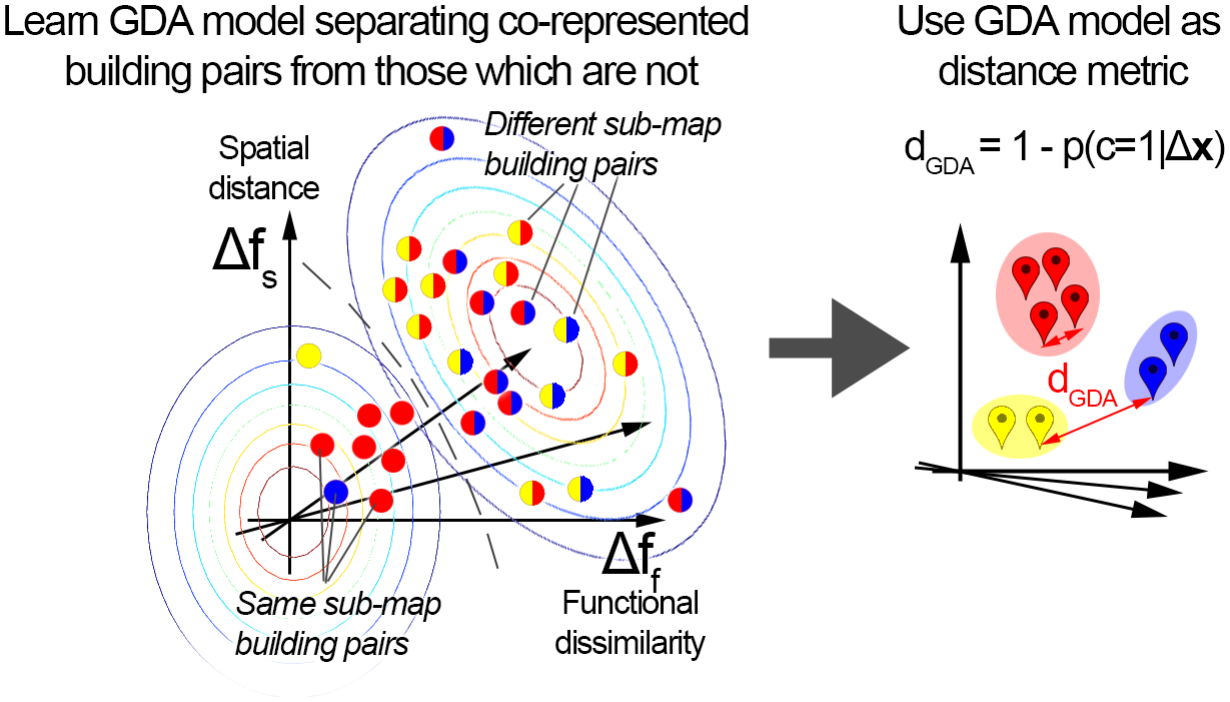
Learning a Gaussian-based non-linear metric. Left: based on a participant’s known map structure, a probabilistic model (GDA) can be trained which can predict the probability of two buildings being co-represented, given their feature differences. Right: These probabilities from a trained GDA model can be taken as similarities and used as the distance metric for a psychological space model. As in the linear models above, map structure predictions for new environments are made by clustering under the learned metric.

Second, our method can naturally incorporate the hypothesis that same sub-map building pair differences should be small (thus located close to the origin), and should be separable from different sub-map building pair differences (these two distributions of pair differences can be naturally modelled using Gaussian distributions)—see Fig 9. Third, most existing machine learning solutions—as well as MDS, used in cognitive psychology to model similarities as distances [53]—need to embed both training map and test map buildings into the same space for model training and testing. This is not possible in our case, because 1) for the features of functional and perceptual similarity, the pairwise similarities across environments are unknown (since subjects only indicate these within each environment, not across environments), and 2) spatial distances might not be comparable across cities or countries (whether two buildings belong to the same representation strongly depends on their geographical distance; but this dependence likely becomes weak or non-existent if they are very far apart).

Finally, for a system additively combining a large number of input signals (as is the case in many neural models of cognition), the Central Limit Theorem suggests a Gaussian-based metric to be plausible; stating that the sum of a large number of variables will converge to a Gaussian distribution.

The proposed **GDA metric** is based on applying GDA (Gaussian Discriminant Analysis) [64] in the difference space Δ**x** (see Fig 8C)—using the set of all training building pairs, a probabilistic (Gaussian-based) model *p*(*c* = 1|Δ**x**) is learned capable of calculating the probability of whether any given pair of buildings are co-represented on the same sub-map, given the differences along various features. We simply define the metric as

$$\begin{array}{c} d\left(x_{1},x_{2}\right)=d_{GDA}\left(\Delta x\right)=1-p\left(c=1|\Delta x\right), \end{array}$$ (4)

where the probability of co-representation is derived using Bayes rule, *p*(*c* = 1|Δ**x**) ∝ *p*(Δ**x**|*c* = 1)*p*(*c* = 1), and the generative densities are modelled using multivariate Normal distributions N (see S1 File for details):

$$\begin{array}{c} p\left(\Delta x|c=1\right)=N\left(\Delta x;\mu_{c},\Sigma_{c}\right) \end{array}$$ (5)

An accurate GDA model (one that correctly separates co-represented and not co-represented building) will ensure that building pairs which are likely to be on the same representation are close, and those which are not are distant, under the metric defined in Eq (4). Just as above, subject map structures are predicted in the testing environments by performing clustering under this subject-specific metric (acquired using data from the training environments).

#### Grouping objects in psychological spaces using clustering

A metric adequately modelling a participant’s representational geometry uniquely characterises her psychological space model. In the case of linear metrics, an object represented by means of its features, $x=(\begin{array}{c} f_{A}\\ f_{B}\\ ... \end{array})$, can be embedded into a new metric space (the psychological space model), in which Euclidean distances correspond to similarities under this metric, by simply weighting each feature by the learned weights of the metric: $p=(\begin{array}{c} w_{a}f_{A}\\ w_{B}f_{B}\\ ... \end{array})$. For non-linear metrics, the embedding is less straightforward, but can be performed by applying multi-dimensional scaling [53] on the full set of objects within the modelled environment, to ensure that the resulting points **p** in the psychological space model have the distances prescribed by the metric.

Subsequently, the presented model performs clustering in this psychological space to generate predicted map structures (groupings of buildings), in accordance with the clustering hypothesis. We employed the DP-GMM (Dirichlet Process Gaussian Mixture Model), from the family of Bayesian nonparametric models, for clustering (see [65] for a tutorial review). Bayesian nonparametric models were successfully employed in categorization models [66] and shown to be psychologically plausible, unifying previously proposed models of human category learning [67] and accounting for several cognitive mechanisms including category learning and causal learning [68], transfer learning [69], and semi-supervised learning [70] in humans. Given that such models give a good account of how humans acquire novel concepts (subsuming prototype, exemplar, and rational models of category learning, among others), and given that they can be seen as probabilistic clustering models, we hypothesized that they might also account for sub-map learning.

DP-GMMs are extensions of Gaussian Mixture Models (GMMs) for an unlimited number of clusters. GMMs are statistical models which aim to partition a set of data points in some space into a number of clusters *C* by fitting *C* Gaussian probability distributions to the data, i.e. adjusting the parameters of these *C* Gaussians such that the probability that the data was drawn from these distributions is maximized. DP-GMMs have the same aim, but also allow inferring the number of distributions (and thus the number of clusters *C*), not just their parameters. In this lies their key advantage compared to most other clustering models: they can be used without prior knowledge of the correct number of clusters (and they can expand by adding new points either to the most likely existing cluster, or to a novel cluster, when observing new data). This process of assigning new data points to clusters by calculating probabilities from distributions optimally fitted to previous data has a lot in common with the basic problem of categorization, which is to identify the category of a new object based on its observed properties and previously observed objects, which is why Bayesian nonparametric models are similar to (in fact, if parametrized accordingly, mathematically equivalent to) multiple psychological models of human category learning proposed in the past [67].

Briefly, the generative DP-GMM model can be defined as follows:

$$\begin{array}{c} \begin{array}{c} \phi_{k}∼Beta\left(1,\alpha_{1}\right)\\ \mu_{k}∼N\left(0,I\right)\\ \Sigma_{k}∼Wishart\left(D,I\right)\\ \pi_{k}∼SBP\left(\phi \right)\\ x_{t}∼Normal\left(\mu_{z_{i}},\Sigma_{z,i}^{-1}\right), \end{array} \end{array}$$ (6)

where SBP stands for the stick-breaking process for generating mixture weights: $\pi_{k}=v_{k}\prod_{j=1}^{k-1}\left(1-v_{j}\right)$, and N denotes the multivariate normal distribution. Data can be generated from this model by first choosing a cluster with probabilities specified by mixture weights: *z* ∼ *Cat*(*π*), and then drawing an observation from the parameters of that cluster *x* ∼ *Normal*(*μ*_*z*_, *Σ*_*z*_). To find a clustering (grouping) of existing data, this generative process has to be inverted. That is, the parameters of a mixture model which characterise each cluster (*π*_*k*_, *μ*_*k*_, *Σ*_*k*_) have to be inferred, for example using variational inference [71]. We have used the *bnpy* Python library for this purpose—see [72] for further implementation details.

Once the parameters are known, cluster memberships *c*_*i*_ of specific points **p** in psychological space can easily be inferred by calculating which of the clusters each point is most likely to belong to:

$$\begin{array}{c} c_{i}\left(p\right)=\underset{c}{\text{argmax}}\;\pi_{c}N\left(p;\mu_{c},\Sigma_{c}\right) \end{array}$$ (7)

Final sub-map membership predictions were derived from the results of the DP-GMM clustering under the metric automatically learned from the subject—buildings predicted to belong together correspond to points in psychological space which are assigned to the same cluster by Eq (7). These predictions were evaluated by calculating prediction accuracies and Rand indices [56]. The former is simply the ratio of perfectly predicted sub-map structures to all subject structures—however, this strict accuracy metric penalizes ‘near misses’ equally to completely wrong structure predictions (e.g. if seven building sub-map memberships are correct, but a single one incorrect, the entire prediction is counted as incorrect; just like completely wrong structures).

Average Rand indices are reported as more fair metrics which provide a continuum between flawlessly correct (*R* = 1) and completely incorrect (*R* = 0) prediction. The Rand index is defined as $R=\left(s+d\right)/\left(\begin{array}{c} B\\ 2 \end{array}\right)$, where *B* is the total number of buildings on a map structure, *s* is the number of building pairs on the same sub-map both in the predicted and actual map structure, and *d* the pairs on different sub-maps both in prediction and in subject data.

## Results

### Overview of the experiments

This section reports the results of four experiments investigating the principles underlying cognitive map structure. Experiment 1 is concerned with the question of whether this kind of structure uncovered by the recall order paradigm is relevant—whether it impacts cognitive performance in other ways than recall orderings —, investigating effects on distance estimation biases, sketch map accuracies, estimated walking times, and planning times in real-world environments well known to subjects.

The plausibility of our central hypothesis—that cognitive map structure arises from clustering—is investigated in the subsequent section, also in real-world environments chosen by subjects themselves. This claim requires buildings that are more similar (closer in psychological space) to be more likely to be grouped together in long-term memory representations, and thus more likely to be recalled together. We report correlations between the probability of buildings being represented together, and proximity in various features relevant to cognitive mapping. We also make between- and across-subject comparisons with regard to feature importances.

Since a good model should be able to make predictions, we proceed to report the predictability of spatial representation structure. We use a clustering model in order to predict map structure, assuming that cluster membership in an appropriate ‘psychological space’ (i.e. under an accurate metric) corresponds to sub-map memberships.

However, feature importances—and thus the metric characterising representational similarities—can vary among participants (see Experiment 1). We utilize three methods to automatically learn subject-specific metrics characterizing the psychological space hypothesized to underlie spatial representation grouping in Experiments 2 and 3. We collect map structures of several different environments from the same subjects in these experiments, using a subset of them to learn a model, and testing its predictions on the remaining subset.

In Experiment 2, participants are asked to learn spatial memories of 3D virtual reality environments. Unlike the other experiments, this approach allows full control over all properties of the stimuli being memorized. Utilizing this flexibility of virtual reality, we report prediction results using clustering, and the decision hyperplane method for acquiring subject-specific metrics, which tackles the challenge of inferring multiple feature importances from few data points by generating the environments such that participants’ subsequent responses minimize the uncertainty of the model regarding the feature importances. After subjects have been queried on a reasonable number of environments, and the model’s uncertainty regarding their psychological space has decreased, they are presented with completely random environments, on which the trained models are tested. We report prediction accuracies both on environments generated such that they minimize model uncertainty (using active learning), and on random environments.

Although virtual reality allows fine-grained control over memorized environments, it is necessarily composed of strongly simplified stimuli and less complex surroundings. To show that the approach of inferring subject-specific models and subsequently clustering objects can also successfully predict cognitive map structure in the much more complex real world, we once again collect data from subjects’ spatial memories of real environments freely chosen by them in Experiment 3. Since the approach of optimally minimizing model uncertainty is infeasible when using uncontrolled real-world memories, we use two more general methods to infer subjects’ psychological spaces, global optimization and a GDA-based metric (see Materials and Methods). Of these, the latter is novel, and the best performing approach on our data. We report prediction results on data excluded from the model training process, substantiating our central hypothesis, and showing, for the first time, the predictability of spatial representation structures on the individual level.

### Experiment 1—Relevance of cognitive map structure extracted from recall sequences

This experiment was conducted to substantiate the recall order paradigm used throughout this paper to infer cognitive map structure. If this paradigm infers something about actual representation structures in spatial memory, then the uncovered structures should have a significant impact on both the speed and accuracy of memory recall for spatially relevant information. To avoid possibly confounding effects of stimulus presentation and memorization, the stimuli used were ones participants had already committed to their long-term spatial memory—the experiment used buildings subjects were already very familiar with and could easily recall information about.

The possible objection that the structures might be induced by the experimental paradigm, and learned by participants during the trials, can be excluded, because of the approximately uniform distribution of the outlier sequences (the first few sequences were not more likely to be outliers than the last few sequences, and no evidence for any learning effects during the real-world experiments could be found in the data—see S1 File for details).

Although data consistent with two of the results presented in this section (the effects of map structure on distance estimation biases and sketch map accuracies) have been observed and published before, this prior work had used significantly fewer subjects than our experiment, and exclusively university students, unlike our participants. [6] had six participants, reporting distance biases and sketch map accuracies; and [8] had twenty eight, reporting only the former.

#### Participants

One hundred and fifty two participants were recruited, consented, and compensated through the Amazon Mechanical Turk (MTurk) online survey system (78 females, 74 males). Participants were required to have at least 95% approval rating on previous MTurk jobs to ensure higher data quality, and all of them were over 18 years of age (as required by the website).

#### Procedure

The experiment was conducted on a website participants could access through MTurk after giving their consent. In the first two questions, subjects were asked to enter the name of a city they were very familiar with, and, subsequently, to pick five buildings they know well. Thus, well-established long-term memories were tested instead of novel stimuli. Subjects were instructed to make sure that they knew where in the city these buildings were located, how to walk from any one building to any of the others, what each building looked like, and what purpose it served. They were only able to proceed past this stage if the website was able to locate all five of the buildings on a geographical map (Google Maps API—https://developers.google.com/maps/—was used to look up the latitude and longitude of each building).

To verify that subjects had indeed formed allocentric spatial representations of the area of the city they had selected, and to allow the analysis of the accuracy of their representations, they were also asked to produce a ‘sketch map’, by dragging and dropping five labelled squares representing their buildings into their correct place using their mouse (Fig 4A, top). No cues or information was provided on the sketch map canvas, just an empty gray surface with five squares labelled according to subjects’ entered building names. Thus, only the relative configuration of the buildings was analysed in this research, after optimal translation, rotation and scaling to fit the placement and size of the correct map as well as possible, by using Procrustes transformation [48].

After the sketch map, subjects performed a seven-trial recall test. In five of the seven trials, they were given a cue or starting building, and were instructed to *‘recall all five buildings, beginning with the starting buildings and the buildings that you think go with it’*, encouraging recall of building names in the order they came to subjects’ mind, closely following the instructions given by [6, 8] and others. In the remaining two, uncued trials, subjects were asked to start with any building they wished. If subjects omitted or incorrectly recalled any of the buildings, they had to repeat the trial (thus, only the ordering changed across trials).

The recall test allowed the experiment software to immediately infer subjects’ map structure using the tree analysis algorithm (see Materials and Methods. Smallest sub-maps—those not containing further sub-maps—were extracted). The next stage of the experiment proceeded based on this structure. Participants were first asked to estimate the time required to walk between four pairs of buildings. Unbeknownst to them, two of the estimations concerned within-, and two of them across-sub-map pairs, in randomized order, and were chosen such that the Euclidean distances in the within-cluster trials were as close as possible to the distances in the across-cluster trials, to mitigate effects of simple distance, as opposed to map structure. After reading the instructions in their own time, subjects were told to estimate and enter the walking time in minutes (the time required to walk from one of these buildings to another) as rapidly as possible. Their responses, as well as their response times (time elapsed between presentation of the pair of buildings for walking time estimation and subjects entering a number and clicking a button) were recorded.

In a subsequent stage, also based on the uncovered map structure, participants had to estimate the distance between four pairs of buildings (Euclidean distance—‘as the crow flies’—as opposed to the walking times of the previous stage). Once again, two within-cluster and two across-cluster pairs were selected such that within- and across-cluster trials differed as little as possible from each other in terms of spatial distance.

Finally, once again in an untimed fashion, subjects were asked to judge the similarities of all pairs of buildings, i.e. $\left(\begin{array}{c} 5\\ 2 \end{array}\right)=10$ pairs, as well as a control pair of one of the buildings to itself, both in terms of visual similarity, and similarity of purpose/function—thus, they had to enter 2x11 similarity judgements. Similarities were judged with the help of 1-10 rating scales, with 1 standing for not similar and 10 for very similar. The two self-similarity judgements were randomly interspersed and verified to avoid subjects rushing the process or entering random values.

Ground truth geographical maps containing participants’ self-chosen buildings were constructed by obtaining latitude and longitude coordinates from Google Maps API, and utilizing an elliptical Mercator projection to obtain x and y coordinates suitable for comparison with subjects’ sketch maps. Euclidean distances between buildings were also calculated based on this projection (as this procedure is more accurate than most alternatives such as the Haversine formula). Finally, path distances as well as ground truth walking times were obtained from Google Directions API, which plans the shortest possible walking route between two buildings along pedestrian paths (which is usually distinct from, and longer than, Euclidean or ‘beeline’ distance).

#### Results

Participants with sketch maps not significantly better than random chance were excluded (using the procedure described in the Materials and Methods section). 86 participants with reasonably accurate survey knowledge of their chosen environments remained (40 female, 46 male). Of these participants, 53 had structure apparent in their recall sequences (20 female, 33 male). The difference in the ratio of structured representations between male (72%) and female (50%) participants is statistically significant at *p* = 0.04 (*U* = 4.39) according to a Mann-Whitney U test. We employed this test here and for a majority of our other significance tests (unless otherwise specified), because the tested variables were not normally distributed according to a Shapiro-Wilk normality test (*p* = 0.00, *W* = 0.63), violating the assumptions behind ANOVA or t-testing. The Mann-Whitney test is a nonparametric test which has greater efficiency than the t-test on non-normal distributions (and is comparably efficient to the t-test even on normal distributions) [73].

To test whether map structure has an impact on other cognitive phenomena, we compared estimations of distance, walking times, and planning times, between pairs of buildings lying on the same representation (within sub-map estimations), and pairs of buildings on different representations (across sub-map estimations). Table 1 reports the results (6 across sub-map and 1 within sub-map distance estimations were excluded, because they exceeded 10*km*, clearly violating the instruction of being within walking distance). Reported correlations are Spearman’s correlation coefficients, here as well as throughout the paper.

**Table 1:** Effects of spatial representation structure on distance estimation, walking time estimation, and response times. All of these estimated magnitudes, as well as response times, are significantly smaller when both buildings are on the same sub-map (i.e. on the same representation) compared to when they are not. Data from 380 pairs of buildings were compared (269 across sub-maps, and 111 within sub-map). Apart from the representation-dependent biases, subject estimations were reasonably accurate (correlation of *r* = 0.40 between estimated and actual Euclidean distance, and *r* = 0.48 between estimated and actual walking time as calculated by Google Maps).

	Actual distance (m)	Estimated distance (m)	Distance bias (Estimated-Actual)	Estimated walking time (min:sec)	Response time when estimating walking time (s)
Within mean Within std	*μ* = 1242, *σ* = 1508	*μ* = 676, *σ* = 1036	*μ* = −574, *σ* = 1825	*μ* = 8 : 43, *σ* = 8 : 23	*μ* = 8.4, *σ* = 6.0
Across mean Across std	*μ* = 1245, *σ* = 1931	*μ* = 1139, *σ* = 1739	*μ* = −146, *σ* = 1703	*μ* = 12 : 45, *σ* = 11 : 36	*μ* = 18.0, *σ* = 92.3
Significance of difference	*p* = 0.109 (nonsignificant), *U* = 12594	*p* = 0.019 (significant), *U* = 12502	*p* = 0.047 (significant), *U* = 11900	*p* = 0.001 (significant), *U* = 11009	*p* = 0.030 (significant), *U* = 13097

In order to avoid effects arising purely from differences in spatial distance, we have queried subjects on the pairs of their buildings (among all possible pairs) which were the least different in spatial distance. In these comparisons, effects purely of spatial distance are unlikely, since distances were not significantly different between within sub-map and across sub-maps estimations (1242*m* and 1245*m* on average)—according to a Mann-Whitney test (*U* = 12594, *p* = 0.11), the difference is not significant.

We have also examined the effect of whether maps were structured on sketch map accuracies. The sum of squared errors (SSE) between the resulting sketch map building positions and the geographical building positions were calculated, and SSEs for all maps with structure (*μ* = 0.305, *σ* = 0.276) were compared to the SSEs for maps without structure (*μ* = 0.370, *σ* = 0.307). SSEs were found to be significantly smaller for structured than for unstructured maps (*p* = 0.019, *U* = 2325), hinting at a correlation between map accuracy and structuredness which can indeed be observed (*r* = −0.17, *p* = 0.04).

Finally, the SSEs between sketch map and geographic map distances were compared for pairs of within sub-maps and pairs across sub-maps, after alignment and normalization. The sketch map distance SSEs within sub-maps (*μ* = 0.607, *σ* = 1.677) were significantly smaller than those across sub-maps (*μ* = 0.916, *σ* = 1.53) according to a Mann-Whitney U test (*p* = 0.023, *U* = 6304000).

#### Discussion

The highly significant differences in the accuracies of sketch maps, distance and walking time estimations, which all depend on whether or not the buildings involved in the estimation are on the same sub-map or on different sub-maps, substantiate the claim that the structures uncovered by this method are indeed relevant, and play a significant role in multiple cognitive mechanisms.

The trends in the distance error biases—distances generally being underestimated within sub-maps compared to across sub-map estimates—match previously made observations using smaller numbers of subjects [6]. The main difference is that this previous work has found underestimation within- and overestimation across sub-maps, whereas our results suggest underestimation in both cases. The negativity (underestimation) of the across sub-map distance estimation errors is statistically significant compared to the null hypothesis that there is zero or positive bias (*p* = 0.03, *U* = 4937).

Both the difference in estimated walking times, and the differences in the response time in this question, are novel results. As opposed to Euclidean distance estimation or sketch map drawing, which can be done by glancing at or recalling a geographical map, accurate walking times are difficult to estimate without actually having explored this environment and being able to plan the routes in question. Subjects need to mentally plan the route and simulate the walk to estimate the time (or to recall the duration of the walk from long-term memory, should the durations of all walks between all possible building pairs be readily memorized by subjects, which is unlikely). The observation that the mean time required to do so more than doubles across sub-maps, compared to within (and that the variance in RTs increases by an order of magnitude) provides additional, substantial evidence for the relevance of map structures—as inferred from recall sequences—to spatial cognitive processes.

### Clustering and features determining map structure

In the Introduction, we have hypothesized that the structure of spatial representations in humans arises from clustering within some psychological space. In this section, we investigate the plausibility of this hypothesis. If this was the case, we would expect the probability of a pair of buildings being co-represented (i.e. represented on the same sub-map) to strongly depend on their ‘similarity’ or distance across various features including spatial distance, with stronger dependencies for spatially relevant features compared to semantic or visual features. These dependencies should be apparent across a population of individuals, even if they assign slightly different importances to each feature (i.e., even if the metrics characterizing their representational geometries differ).

We would also expect several features to play a role, since distance alone is insufficient to explain previous results [6, 8]. We would expect the relevance of each feature to be apparent from its influence on map structure, measurable by the correlation between co-representation probability (the probability that two buildings are co-represented on the same sub-map) and the distance in this feature. Finally, we would expect large inter- but small intra-subject variability in these correlations, i.e. stable feature relevances within subjects which are not necessarily generalizable across subjects, analogously to psychological spaces for concept representation [43, 74].

We investigate several features listed below, motivated by hints in the literature that they might play a role in the representation structure of object-location memory.

1. Remembered distance, i.e. the distance on subjects’ sketch maps
2. True Euclidean distance based on geographical maps
3. Path distance (or ‘city-block’ / ‘Manhattan’ distance), since recent brain imaging evidence suggests that the hippocampus—a spatially relevant brain region—represents both Euclidean and path distances [75]
4. Boundaries in the environment (such as rivers, cliffs, city walls, etc.)—based on neuroscientific and behavioral evidence that boundaries play an important role in spatial memories [5, 37]
5. The number of streets separating a pair of buildings (intersecting with a straight line connecting these buildings)
6. The sizes of separating streets; that is, whether these streets could easily be crossed (whether or not they were highways/motorways/primary roads which are difficult for pedestrians to cross)
7. Visual similarity (as indicated by participants), since clustering by perceptual properties has been reported [20], and vision has been suggested to be vital to spatial representation [76],
8. Functional similarity, or similarity of purpose, as indicated by participants—because action-based similarity has been claimed to have an effect on spatial memory [38], and also because of the importance of action-related roles within the influential grounded cognition paradigm [77].
9. Phonetic and morphological similarity of building names. The main motivation behind including these features was to investigate any possible interference on the structures inferred from recall sequences caused by verbal short-term representations. Subjects might employ some short-term representation strategy to complete the recall trials more rapidly—instead of recalling from long-term spatial memory -, for example subvocal rehearsal loops (articulatory loops). Including phonetic and morphological similarity features helps measure the effect of such verbal strategies.
  (Phonetic similarities have been determined using the Double Metaphone [78] phonetic encoding algorithm, since it accounts for irregularities in multiple languages, not just English. This was important for names from non-English speaking countries—see S1 File. Morphological similarities were calculated based on recent work by [79]. For building names consisting of multiple words, the sum of the respective phonological or morphological similarities of the constituent words was used.)

The first six of these features—remembered, Euclidean and path distance, and boundaries, separating streets, and crossable streets—were obtained based on geographical data available online. Most such geospatial ground truth data used was obtained using Google’s publicly available Maps API, with the exception of boundaries in the environment, and crossable streets (whether separating streets were difficult to cross)—these two features were obtained from Open Street Maps (OSM) through their publicly available API called Overpass (http://overpass-api.de/). As in all experiments in this paper, ground truth maps and distances are based on an elliptical Mercator projection of latitudes and longitudes obtained from Google Maps API, except for path distances and walking times which were queried from Google Directions API.

All features were converted into distances / dissimilarities before subsequent analysis. Similarity features, such as visual, functional, phonetic and morphological similarities, were subtracted from the maximum value possible for that feature to obtain corresponding dissimilarities.

#### Participants, Materials, and Procedure

The clustering hypothesis introduced in the Introduction implies that buildings closer together in psychological space are more likely to be represented on the same sub-map in participants’ spatial memory. Data from Experiment 1 as well as Exp. 2 and Exp. 3 A and B were analysed with regard to the plausibility of the clustering hypothesis, as well as the underlying features determining map structure. Thus, the participants, materials and procedures for data collection were exactly the same as in those experiments, following the recall order paradigm described in the Materials and Methods section and in S1 File.

All Figures in this Section are split into four parts, for Experiment 1, Exp. 2, and conditions A and B of Experiment 3. We report results separately, since there were slight changes in procedure. Briefly, Exp. 2 was conducted in three-dimensional virtual reality environments, whereas the other experiments used subjects’ established real-world spatial memories. Furthermore, cues were presented verbally in Exp. 1 and 2 and spatially, highlighted on sketch maps, in Exp. 3 (Exp. 3B also used 8 buildings, unlike the 5 used in the other experiments). Finally, Experiments 2 and 3 tested spatial memories of several different environments, in order to facilitate acquiring a model and testing its predictions, whereas Exp. 1 did not.

**Fig 10:**
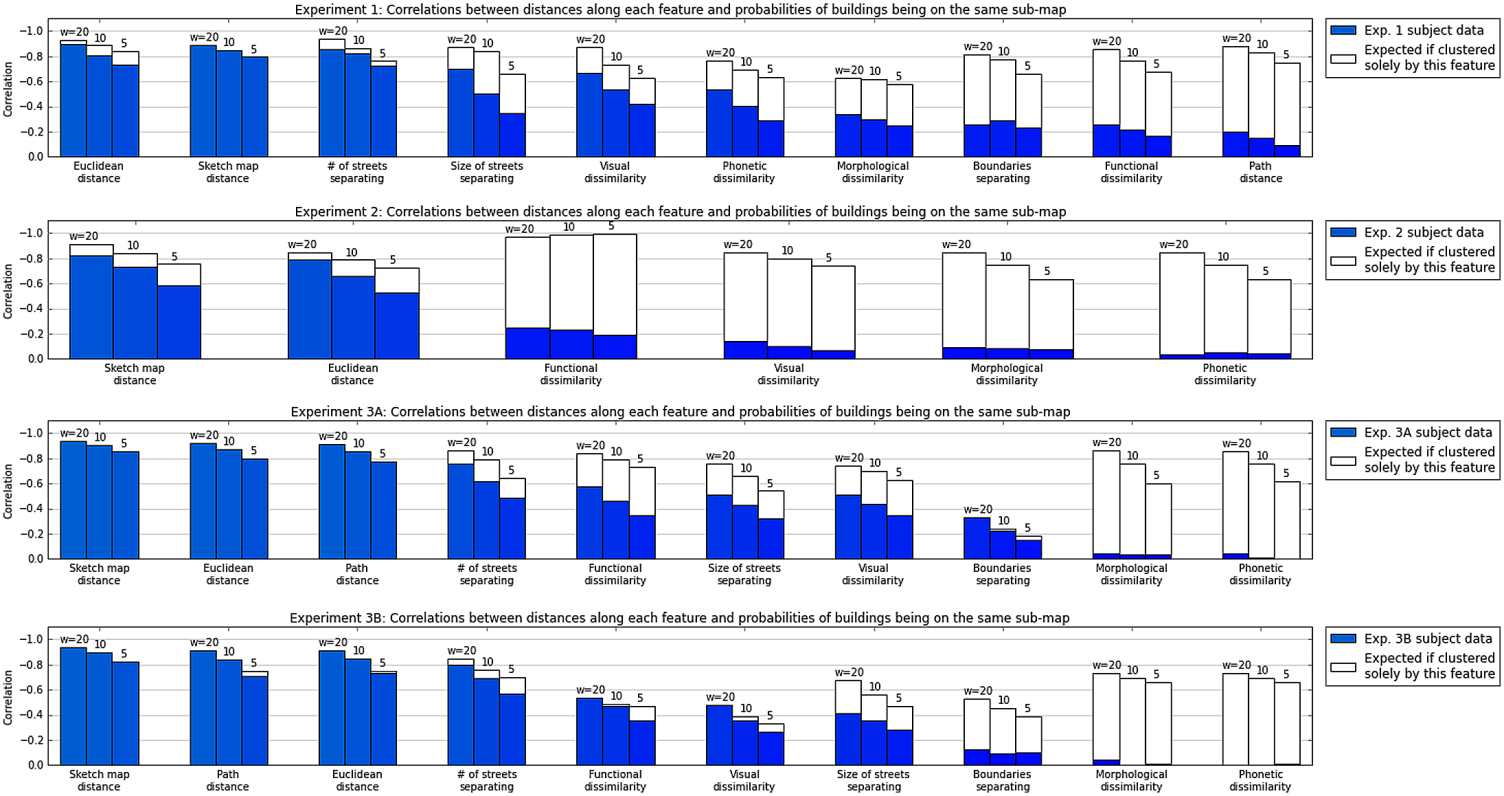
Correlations between probabilities of being on the same sub-map, and distances along each feature, for pairs of buildings in Experiments (from top to bottom): 1, Experiment 2 in virtual reality (therefore lacking geospatial features), and 3A, 3B. Correlations are reported separately for each feature. The three bars per feature show results at three different window sizes *w* used for calculating co-representation probabilities (higher *w* lead to less noisy probability estimates through smoothing, resulting in higher correlations). Empty bars show levels of correlation that would be expected if maps were clustered according to the single respective feature only.

To test the clustering hypothesis, we investigated the co-representation probabilities of pairs of buildings belonging on the same sub-maps, and each of the features listed above. In particular, the correlations *r*_*f*_ for each feature reported in Fig 10 are the Spearman’s rank correlations between the differences **Δx**_**f**_ for that feature *f* (e.g. the number of streets separating two buildings), and the co-representation probabilities **P**_**f**_, i.e. the probabilities of pairs of buildings belonging on the same sub-maps. Simply put, the likelihood of co-representation at a specific distance equals the ratio of the number of co-represented pairs divided by the number of all pairs within some small window *w* close to this distance.

Let **Δx**_**f**_ = (Δ*x*_*f*,1_, …, Δ*x*_*f*,*n*_) be the sorted sequence of differences in feature *f* for all pairs of buildings across all participants and all environments, such that Δ*x*_*f*,1_ is the smallest such difference. Furthermore, using the same sorted ordering of building pairs, let $C_{f}=\left(c_{f,1},...,c_{f,n}\right)$ contain *c*_*i*_ = 1 if building pair *i* belongs to the same sub-map, and *c*_*i*_ = 0 otherwise, for all building pairs (and sorted such that *c*_*f*,1_ expresses whether the buildings closest in feature *f* are consistently recalled together).

Then, we can define co-representation probabilities **P**_**f**_ = (*p*_*f*,1_, …, *p*_*f*,*n*_) for each feature using a moving average of the sorted co-occurrences: $p_{f,i}=1/w*\sum_{j=i-\lfloor w/2\rfloor }^{i+\lfloor w/2\rfloor }c_{f,j}$ (for example, if *w* = 3 and out of three building pairs with distances 95*m*, 100*m* and 105*m* two were represented on the same sub-map, then the probability of co-representation at 100*m* would equal *p* = 2/3). The correlations reported below were calculated between these co-representation probabilities **P**_**f**_, and the feature differences **Δx**_**f**_, both in the same order, sorted by the feature differences. In cases where there were too few data points to estimate **P**_**f**_, the biserial correlation coefficient [80] was calculated between the binary co-representation values $C_{f}$ and feature differences **Δx**_**f**_.

#### Results

Fig 10 provides an overview of the correlations of these features with the probabilities of co-representation on the same sub-map, reporting Spearman correlations *r*_*s*_(**P**_**f**_, **Δx**_**f**_) for each feature *f*. The correlations are reported for three different window sizes *w*, to show that the high correlations are not artefacts of a particular window. Supporting the central hypothesis of this paper, which implies that buildings closer together in psychological space are more likely to be on the same sub-map, we indeed found strong correlations between multiple of the above-mentioned features, and the probability that two buildings were clustered together.

The Figure also shows the correlations that could be expected if participant’s map structure had arisen from clustering by just that one feature (empty bars in Fig 10)—i.e. the correlations that would have been observed had participants 1) used clustering to structure their maps, and 2) used only distances within one respective feature for this clustering. These expected correlations were calculated using the same participant data; but artificially structuring the subject map—using clustering along one respective feature—instead of using subjects’ sub-map memberships. Gaussian mixture models (GMMs) [81] were used for the artificial structuring, just like for prediction in the computational models described below, since they are more psychologically motivated than other clustering algorithms (see Materials and Methods).

**Fig 11:**
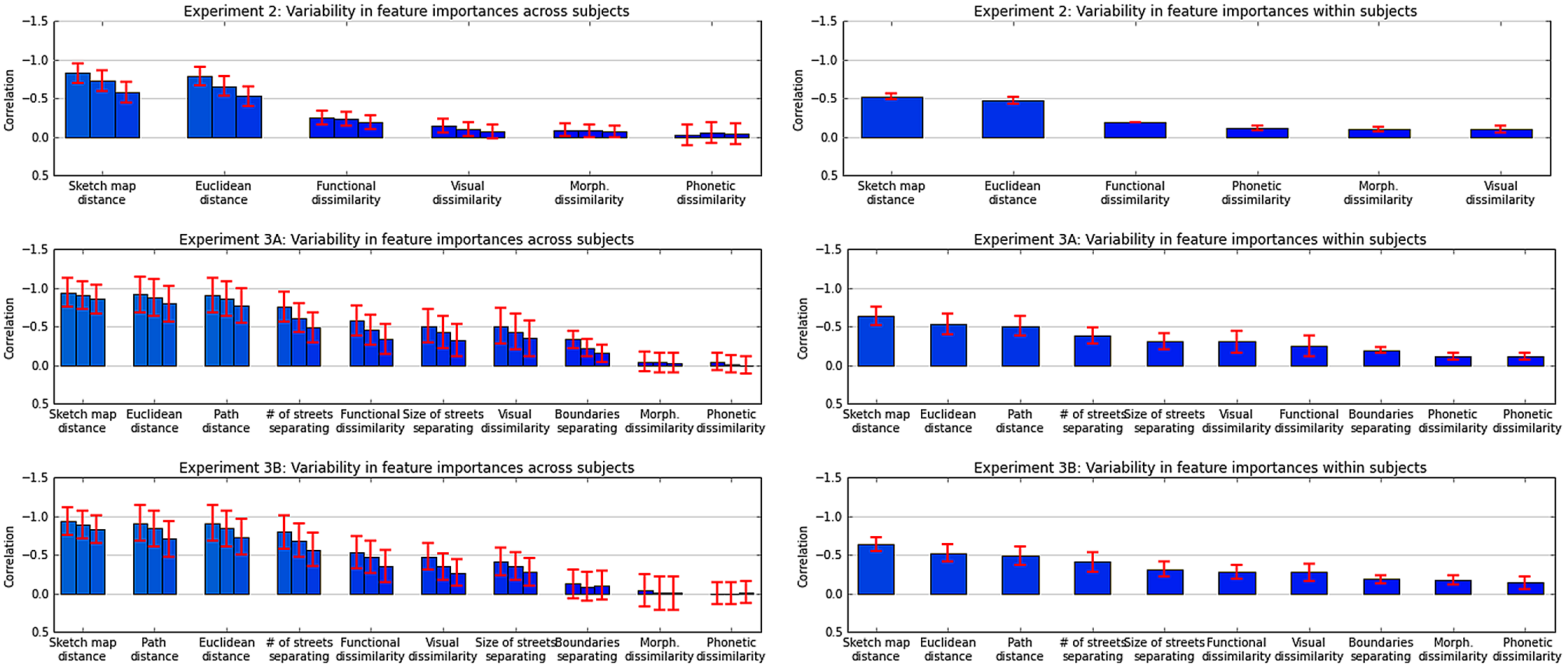
Variability of features influencing cognitive map structure. Feature variabilities across all subjects (left) and across map structures of individual subjects (right) are shown, plotted as error bars on each average feature correlation. Top: Bottom: Feature variabilities in the test trials of Experiment 2. Middle: Feature variabilities in Experiment 3A. Bottom: Feature variabilities in Experiment 3B.

Next, we have investigated the variability of the reliance of these features within and across subjects; i.e. whether the same features were used by—and whether they were similarly important for—all subjects, and whether they were the same for individual participants in different environments. Fig 11 shows the standard deviations of the co-representation correlations of these features, across subjects (left panels) and within subjects, i.e. across the maps of individual subjects (right panels), averaged over all subjects.

Specifically, the standard deviations of the biserial correlation coefficients [80] between the binary co-representation values and feature differences $r_{pb}\left(C_{f},\Delta x_{f}\right)$ are reported for all error bars in the plot. (Biserial correlation with the binary vector indicating same or different sub-map pairs was used, instead of calculating probabilities and using continuous correlation, because the numbers of available within and across sub-map pairs of buildings for a specific map of a specific participant were frequently below the window sizes used for estimating co-representation probabilities in Fig 10, preventing the estimation of these probabilities). For the within-subject plots (right panels), the magnitude of the bars is also calculated using biserial correlation, for the same reason—there being too few within-subject building pairs for the moving average-based probability calculation.

Finally, according to Shapiro-Wilk normality tests, none of the distributions of feature correlations are normally distributed (all p values for all features are many orders of magnitude less than 0.01). Rather than most subject structures arising from these features weighted in the same fashion, or from feature correlations concentrated around a most common value, the particular strengths of the influences on participants’ representation structures seem to be irregularly distributed. However, they are significantly more consistent across individual participants’ map structures (Fig 11 right) than across all participants (Fig 11 left).

#### Discussion

The strong dependence of co-representation probability on distance along various features (Fig 10) provides strong evidence for the plausibility of the clustering hypothesis in participants able to learn allocentric cognitive maps. Furthermore, confirming intuitive expectation, spatial features show a much stronger influence on map structure than other, non-spatial dimensions.

Fig 11 shows that there is a large amount of variability in the importance of different features to various subjects. This spread is significantly less across the map structures of individual participants (Fig 11 right) than across all participants. Thus, although collecting a high enough number of map structures to reliably infer subject-specific feature importances presents several practical challenges (see next section), doing so is unavoidable for predicting spatial representation structures.

It is important to point out that correlation with co-representation probability alone is not a sufficient metric for describing the influence exerted by a feature on cognitive maps. There might be indirect causation or a common cause, or deceptively low correlations due to sparse data (for example, very few natural boundaries are present in most cities, which causes low correlations despite their importance according to the results below), or other reasons for correlation not translating to causation.

For this reason, to what extent different features facilitate the prediction of individual map structures is a more meaningful measure of their importance in the cognitive map structuring process. The following sections report prediction results, both in automatically generated virtual reality environments (Section 2) and in real-world environments freely chosen by subjects (Experiment 3).

### Experiment 2—Predictability of map structure in virtual reality environments

This experiment investigated the question whether the clustering hypothesis allows robust advance prediction of participant map structures. Because of the observation that feature importances vary greatly across subjects, but less for individuals (Fig 11), it was designed to first learn these per-subject importances, before producing predictions using a clustering mechanism. This process was inspired by *active learning* [57], a field in machine learning which allows algorithms to choose the data from which they learn, thus facilitating better performance with less training data. This latter point is crucial for our experimental paradigm—as inferring the representation structure of even small environments with few buildings requires several full recall sequences, there is a practical limit on how many structures per participant can be produced—thus, this limited budget of data should be used in a fashion close to the statistical optimum. Optimally reducing model uncertainty using active learning is one possible approach towards this objective.

#### Participants

Fifty students at the University of Manchester (compensated by vouchers) participated in the experiment. 38 subjects who did not produce sketch maps significantly better than random chance in at least 50% of all training trials were excluded, leaving 12 subjects whose data was analysed. Participants were recruited and tested at the University of Manchester (instead of online) primarily because the setup required a modern PC equipped with a graphics card to run the experiment smoothly. A further reason was the need to ensure that the setup was equivalent across subjects (e.g. screens were of the same size and quality, all subjects used a mouse and not a touchpad, etc.).

#### Procedure

After giving their consent and reading instructions, participants completed 20 trials—15 ‘training’ trials which were used for training the model, and 5 ‘testing’ trials which were used for verifying the predictions of the computational model. In total, the experiment took about 1.5 hours on average. Each trial was set in a unique environment consisting of a horizontal ground plane, featureless sky, and 5 buildings. All buildings used the same 3D model and thus had equal measurements, but could vary in colour, in function (being labelled as either shops or houses) and in distance; and could have different labels (e.g. coffee shop, John’s house).

Both trial types followed the same sequence. First, participants could freely explore the environment, and were asked to memorize the positions and names of all buildings in it. In this memorization phase, they were also asked to deliver a package from one of the shops to one of the houses. This task served the dual purpose of forcing subjects to do a minimum amount of exploration, and, additionally, to make the functional distinction between shops and houses more meaningful. After the memorization phase and the delivery task, the environment vanished, and participants’ spatial memory was tested, by asking them for 1) a sketch map, produced by dragging and dropping labelled squares into their correct places, and 2) seven recall sequences, 5 cued, and 2 uncued.

The first 15 ‘training trials’ each contained two distant groups of two buildings in close proximity, and a ‘middle’ building somewhere between these two groups. Both buildings in each group always had the same colour and function, and there was always one group containing two shops and a second group containing two houses. The middle building was intended to be represented together with one or the other group by subjects, depending on its distance and similarity to the groups. Data from trials in which the middle building was not clearly co-represented with one or the other group, or in which no groups were present, were excluded. All subjects showed the two groups in their structure, and co-represented the middle building with one of them, in more than half of their trials. The 16% of the trials in which this was not the case were excluded from further analysis.

In the first 7 of the included trials, the colours, functions, and distances of the groups and the middle building were generated randomly, ensuring only within-group consistency of colour and function and that buildings within groups were closer than the distance between the groups, such that they unambiguously formed clusters.

After the 7th training trial and all subsequent training trials, a ‘decision hyperplane’ was calculated using logistic regression, which separated all middle buildings into two groups, those belonging to the shop cluster, and those belonging to the house cluster. (The initial seven trials were required to initialize a reasonably accurate hyperplane to generate further environments from—seven being the minimum number required for reliably good performance in preliminary simulations). This decision hyperplane facilitated the generation of the remaining 8 training trial environments. For each trial after the 7th, the two groups were again generated randomly, but the middle building was parametrized such that the uncertainty regarding subjects’ feature importances was minimized. To achieve this, the parameters of the new middle building were drawn from the region of the currently calculated decision hyperplane, since this is the region in which the model is least certain as to where buildings should be assigned (see Materials and Methods). Formally, this is equivalent to active learning [57] with uncertainty sampling [59] in machine learning. Each of these remaining 8 training trials maximally reduced the model uncertainty regarding feature importances.

Finally, participants completed 5 ‘testing’ trials, going through the same procedure of memorization, delivery task, and producing a sketch map and recall sequences. These testing trials were generated randomly, without any restrictions on building parameters, not even the restriction of there needing to be clusters defined in any way along any of the features (however, testing environments were required to contain at least one shop and one house). They were used to test the predictions of the computational model.

#### Results

We included all features described above in the following analysis, except for the four geospatial features not relevant in our simple virtual reality environment (path distance, natural boundaries, number of intersecting streets, whether they could be easily crossed). See Fig 10 for the correlations of these features with co-representation probabilities, and Fig 11 for the across- and within-subject variances of these correlations.

Above, we have introduced a method to infer participants’ feature importances for clustering, based on the inference of a decision hyperplane describing at which point in feature space subjects stop assigning a middle building to one sub-map and start assigning it to another. With this method, we have both components of a predictive model of cognitive map structure: 1) subjects’ psychological spaces, spanned by a set of features and feature importances, as inferred by the decision hyperplane approach, and 2) a clustering algorithm. We chose DP-GMM as the clustering algorithm, given its substantial advantage of being able to infer the number of sub-maps automatically, and motivated by its success in other psychological models.

**Fig 12:**
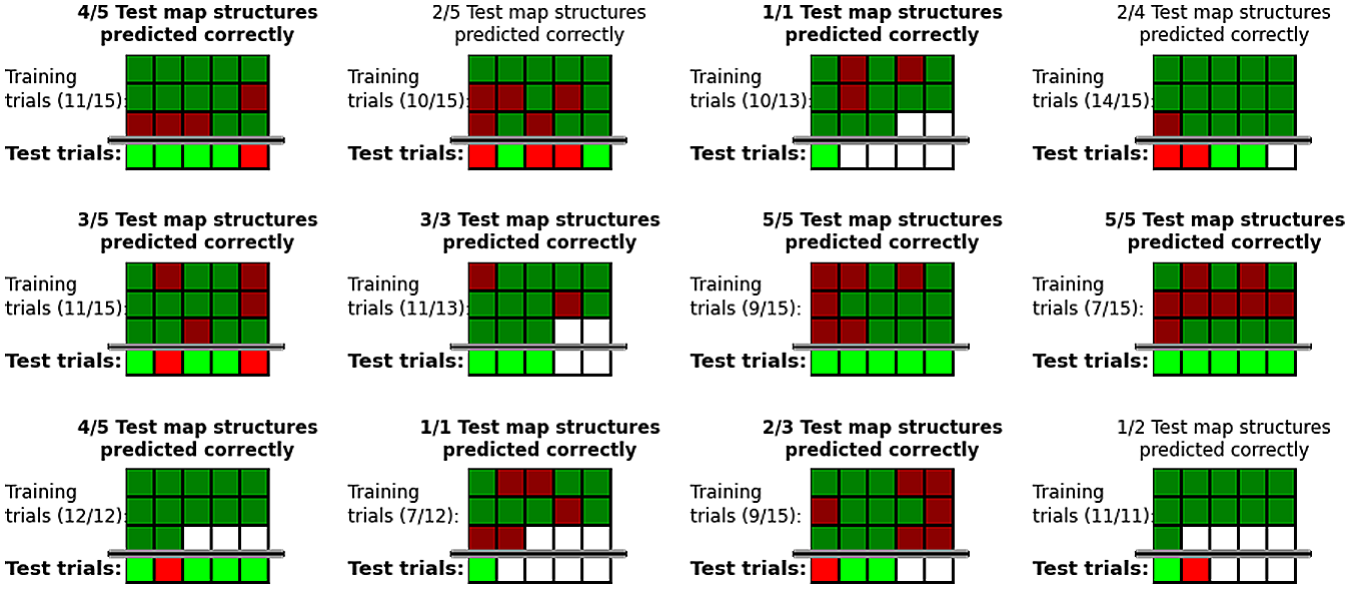
Results of a predictive clustering model using subjects’ feature importances, learned using the decision hyperplane approach. Each sub-plot reports all prediction results for one subject, using green cells for correct predictions, red cells for incorrect predictions (one or more buildings grouped to the wrong sub-map), and white cells for subject maps either not better than random chance or without apparent structure. Top 3 rows in each subplot show results on the training trials (dark colours), and the 4th, bottom row shows the prediction accuracies on the test trials (bright colours). On average, 75% of all test map structures could be predicted correctly (green cells). For comparison, the probability of prediction by random chance is 0.4% for two sub-map and 3.1% for one sub-map structures.

Fig 12 shows the results of this predictive model on all participant cognitive map structures (20 per subject; 15 training maps used to infer feature importances, and 5 testing maps used to verify model predictions). Prediction can be incorrect on training trials, because feature importances are being inferred using the decision hyperplane approach without taking into account the clustering algorithm and its idiosyncrasies (see red cells of the first 3 rows). After inference of feature importances and running the clustering model within this feature space, 73.5% of the training map structures could be predicted.

The interesting part of Fig 12 is the bottom row of each sub-plot, which contains the advance predictions of the model on randomly generated environments it was not trained on and not confronted with prior to making the prediction. On average, **75.0% of all test map structures could be predicted correctly in advance** using the decision hyperplane method and DP-GMM for clustering; and the majority of map structures could be predicted for all subjects except for one.

Note that this is a strict accuracy metric—if the model predicts four out of five building sub-map memberships correctly, but a single one incorrectly, the entire prediction is counted as incorrect. The Rand index [56] is a more comprehensive metric, providing a number between 1 (flawless clustering) and 0 (all cluster memberships incorrect). The **average Rand index of predicted vs. actual test map structures was 0.83** in this experiment, meaning that for 83% of the pairs of buildings, it could be correctly predicted whether or not they belong to the same sub-map in participants’ spatial memory (according to their recall sequences).

If using the same DP-GMM model with feature importances inferred from co-representation correlations instead, the prediction accuracy drops to 59.1% on the testing maps, with an average test-map Rand index of 0.75, indicating that the decision hyperplane approach is better suited to uncovering feature importances than just using correlations.

Since each environment contained five buildings, there could be up to two sub-maps, and the clustering process could be framed as assigning one of three values to each building—member of sub-map #1, or of sub-map #2, or a single-building cluster (sub-maps with only a single building were excluded from participant data, for reasons explained in the Materials and Methods section; however, if the model produced single-building clusters, these were not excluded from the model predictions, but instead counted as mistakes). Thus, the baseline probability of randomly coming up with the correct clustering is, on average, (1/3)^5^ = 0.4% for map structures with two sub-maps, and (1/2)^5^ = 3.1% for structures with one sub-map of unknown size. In this experiment, 14 subject test map structures contained two sub-maps, and 30 structures one sub-map.

#### Discussion

The observation that a large majority of subject map structures can be predicted in advance using a clustering model, together with an appropriately scaled feature space, provides further support for the clustering hypothesis. The improvement of prediction accuracy from 59.1% to 75.0% (and Rand index from 0.75 to 0.83) when using the decision hyperplane approach to infer feature importances, instead of just using co-representation correlations, suggests that this approach is more suitable to uncover the psychological spaces in which the clustering takes place.

However, the present approach has some shortcomings. First, it is only applicable to controlled environments—thus, investigating participants’ past long-term memory structures requires different methods (see next section). Second, the fact that calculating feature weights from a decision hyperplane does not take into account the actual model generating the predictions (in this case, the DP-GMM). Finally, the approach assumes linearity, i.e. that the surface separating buildings co-represented with one or the other sub-map is a linear hyperplane (as opposed to a non-linear surface). These shortcomings are reflected in the sub-optimal performance of the model on the training trials in Fig 12. Although a model should be able to fit its training data well, the performance on training trials (73.5%) and testing trials (75%) is not statistically significantly different.

The next section uses two different approaches which are not subject to these shortcomings (introduced in the Materials and Methods section), one learning the optimal feature weights for the employed clustering model using global optimization, and the second lifting the linearity assumption. Both of them have the additional advantage that they do not require controlled environments.

### Experiment 3—Predictability of cognitive map structure in the real world

In this experiment, real-world buildings well known to participants were used (similarly to Exp. 1). Apart from providing additional evidence for the clustering hypothesis by showing that cognitive map structures in real-world environments can be predicted using a clustering model, this section also validates the two generally applicable ways of acquiring subject-specific models (the linear metric learned passively by means of global optimization, and the GDA metric) introduces in the Materials and Methods section. The same features introduced above were used in this experiment. In addition, combinations of features (pairwise products of pairs of features) were also included, in order to allow the learned metric to capture dependences between features.

#### Participants

In total, data from 73 participants was analysed in this section. Subjects unable to produce at least two sketch maps significantly better than random chance, with structure apparent from their recall sequences for at least two maps, were excluded, as at least two map structures were required to have both a training and testing map.

In Experiment 3A (which asked for 5 environments with 5 buildings each), out of 81 participants, 54 had at least two better-than-random and structured maps. In Experiment 3B (which asked for 3 environments with 8 buildings), out of 30 participants, data from 19 were analysed. Participants were recruited, consented, and compensated through the Amazon Mechanical Turk online survey system, and were required to have at least 95% approval rating on previous jobs to ensure higher data quality.

#### Procedure

The procedure was similar to the one used in Experiment 1. This experiment was also conducted on a website participants could access through MTurk after giving their consent. Unlike 1, this experiment consisted of multiple trials (5 in condition A, 3 in condition B), each trial following an equivalent procedure but asking for a completely different set of buildings, possibly in a different city. Subjects took between one and 3.5 hours to complete this repeated trial experiment (this includes possible breaks, since the experiment was performed online in participants’ homes, unsupervised, and the experiment was not timed).

In the first questions of each trial, subjects were asked to pick a number of buildings they know well—5 in condition A, and 8 in condition B (thus, in total, 25 buildings had to be recalled for the 5 trials of condition A, and 24 for the 3 trials of condition B). Thus, well-memorized long-term memories of real-world environments were tested instead of novel stimuli in virtual reality. Subjects were instructed to make sure that they know where in the city these buildings are located, how to walk from any one building to any of the others, what each building looks like, and what purpose it serves.

The subsequent questions of each trial required subjects to produce a sketch map, and to perform a recall test consisting of 7 recall sequences in condition A, and 10 in condition B (in both cases, as many cued sequences as there were buildings on the maps, and two additional uncued sequences). Subjects followed the same instructions as in Experiment 1; the crucial difference being that instead of presenting cues verbally by writing out the name of the cue building, cues were presented visually (cue modality was changed to mitigate the strong effects of phonetic and morphological similarity in the prior experiments, presumably due to articulatory rehearsal strategies). That is, the participants’ own sketch maps were displayed again (showing only their remembered building positions, without any names or labels), and the cue building was highlighted using a thick border and green colouring. In the final question, subjects were asked to judge the similarities of all pairs of buildings, i.e. $\left(\begin{array}{c} 5\\ 2 \end{array}\right)=10$ pairs in condition A and $\left(\begin{array}{c} 8\\ 2 \end{array}\right)=28$ pairs in condition B, as well as a control pair of one of the buildings to itself, both in terms of visual similarity, and similarity of purpose/function (using 1-10 rating scales as before).

#### Results

**Fig 13:**
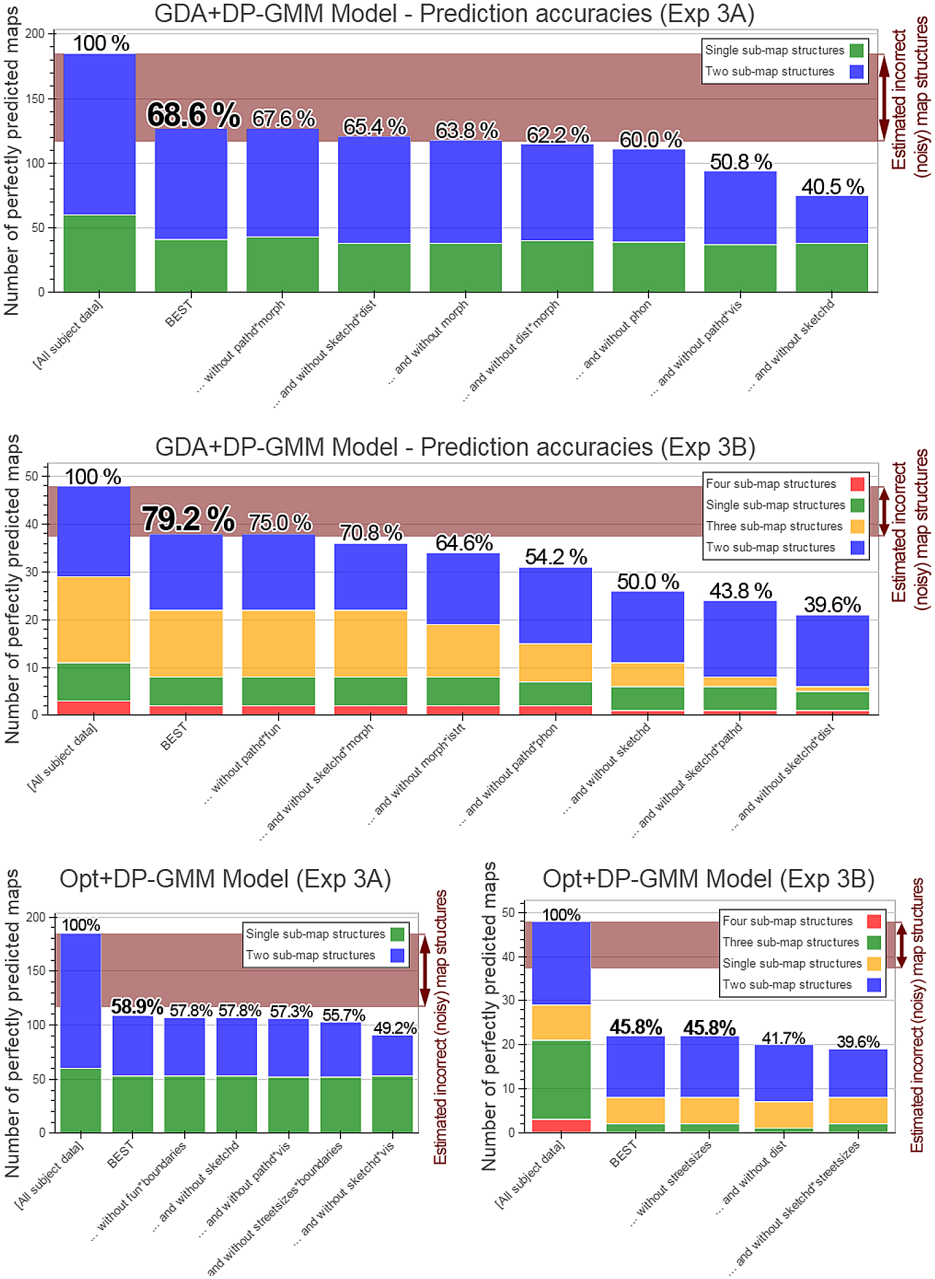
Prediction accuracies. First bars show the numbers of all subject map structures (and, within them, the structures containing the specified numbers of sub-maps). Top: results in condition A. The second bar shows the accuracy for the best feature set (Euclidean, path*morphological, sketch map, path*visual, phonetic, Euclidean*morphological, morphological, sketch map*Euclidean distances); bars 3-8. show accuracies when successively removing the last feature. Middle: results in condition B. The second bar shows the best accuracy (Euclidean, path*functional, sketch map*morphological, morphological*separating streets, path*phonetic, sketch map, sketch map*path and sketch map*Euclidean distances); bars 3-8. accuracies when successively removing the last feature. Bottom: accuracies of the optimization-based metric.

Fig 13 shows prediction accuracies (the ratio of perfectly predicted map structures to all subject map structures) using DP-GMM clustering and GDA subject-specific model learning. The best possible set of features and feature combinations was estimated from the training data, using a greedy search approach—starting with a single feature (Euclidean distance) and then iteratively adding the feature which brings the clustering prediction closest to participants’ actual groupings; repeated until either all features are included or the clustering prediction accuracy stops increasing. Using the best possible set of features shown to the model, **68.6%** **of the 185 subject map structures with 5 buildings of Experiment 3A** (with up to two sub-maps per structure), and **79.2%** **of the 48 subject map structures with 8 buildings of Experiment 3B** (with up to four sub-maps per structure) **can be predicted accurately**, such that every single predicted sub-map membership is correct for these percentages of test maps.

**Average Rand indices for these models are 0.87 for condition A and 0.95 for condition B**, which means that even the structures which are imperfectly predicted, causing a lower than optimal prediction accuracy, are highly similar to the correct structures (co-represented building pairs are predicted correctly in 87% in condition A and 95% in B). Note that the prediction accuracy of the best model is statistically indistinguishable from the estimated maximum possible prediction rate (calculated above based on simulating distractions by random swapping). This suggests that the proposed novel GDA-based method does well at learning subject feature spaces, and that the subsequent clustering model, based on a previously proposed Bayesian model of human category learning, can infer the sub-map memberships and numbers accurately.

Fig 13 also shows the numbers of sub-maps contained in participants’ structures. In general, the prediction task can be seen as assigning one of *K* + 1 values to each building, where *K* is the maximal number of possible sub-maps (single-building clusters are also possible, hence the increment by one). Thus, the baseline probability of randomly coming up with the correct clustering is, for condition A, (1/3)^5^ = 0.4% for map structures with two sub-maps, and (1/2)^5^ = 3.1% for structures with one sub-map. For condition B, this baseline expected random clustering accuracies are several orders of magnitude lower (2.5 ∗ 10^−4^%, 1.5 ∗ 10^−3^%, 1.5 ∗ 10^−2^% and 0.3% respectively for *K* = 4, 3, 2 and 1).

The model accuracies when successively removing particular features (bars from left to right in Fig 13) provide an additional measure for how important these features were, aggregated over all subjects, and measuring importance in a causal fashion, since this is a predictive model. The most important features were those which caused the greatest drops in accuracy upon their removal. In condition A, two features are significantly more important than the rest—sketch map distance and the product of path distance and visual similarity -, whereas the importances are similar in condition B, with a slightly larger accuracy drop caused if omitting sketch map distance. In both conditions, about 2 out of 5 map structures can still be predicted when using solely Euclidean distance.

The bottom row of Fig 13 shows prediction accuracies obtaining using the same procedure, but the passive global optimization-based metric learning method instead of the non-linear GDA metric (see Materials and Methods). The GDA-based method significantly outperforms global optimization, suggesting that the Gaussian assumptions are more reasonable than the linearity assumption made by the other methods.

The strong influence of sketch map distances raises an additional question regarding predictability of cognitive map structures—is advance prediction possible without asking the subject anything (other than a list of buildings he knows)? To investigate this question, we have run the predictive model on data from which visual similarities and sketch map distances were removed, i.e. solely on data which can be derived from the list of subjects’ buildings and geospatial data sources such as Google Maps API.

Subjects’ functional similarities were also removed from this data, and replaced by an objectively calculated measure of functional relatedness. Specifically, we used the Jaccard similarity metric on lists of building types from Google Places API. This API can return a list of known building types when queried—see https://developers.google.com/places/supported_types for a list. Usually buildings have several applicable types, ranging from specific to general, e.g. ‘meal takeaway’, ‘restaurant’ and ‘food’ for McDonalds. The Jaccard index (JI), defined as the ratio of the size of the intersection to the size of the union of two sets, measures how many items in these type lists match between two buildings, as a proxy for their functional similarity. (For example, *JI* = 0.5 between the McDonalds example and a building with types ‘bakery’, ‘restaurant’ and ‘food’). The objective functional similarity metric thus obtained does reflect subjects’ own judgements—the correlation between them is *r* = 0.66—but is somewhat different, since it does not reflect subject idiosyncrasies, and is also free of noise or biases.

Using GDA for subject-specific model inference, and using these features which are all known a priori—derivable from the subject building lists and public geospatial databases -, 75% of map structures can be predicted in advance for condition A (Rand index: 0.91), and 63.4% in condition B (Rand index: 0.88).

Finally, we have attempted to predict subjects’ cognitive map structures without learning subject-specific models at all, by trying to infer a psychological space common to all subjects, and clustering within this space. Inferring someone’s spatial representation structure without knowing anything about them would have great advantages for robotics applications and geographical planning and map design, among other fields (see Discussion). The resulting prediction accuracies (and Rand indices) for condition A and B were 41.7% and 60.2% (and *RI* = 0.76 and 0.78) respectively. In accordance with the individual psychological space hypothesis and with the results in Fig 11, the model performs significantly worse when not allowed to learn subject-specific feature spaces. However, even these impoverished models can predict whether or not two buildings are co-represented on the same sub-map in more than 3 out of 4 cases.

Table 2 summarizes the prediction accuracies and Rand indices of the best models in both condition 3A and 3B, in all three above-mentioned settings (using all features, or only features which do not require asking for similarities or sketch maps, or using a general, ‘one size fits all’ model requiring no questions at all).

**Table 2:** Prediction accuracies (and Rand indices) in Experiment 3, for all features and subject-specific GDA+DP-GMM model (second column), for features known a-priori, without having to ask subjects to rate similarities or draw sketch maps (third column), and finally using a subject-general model, without learning subject-specific feature weights. Rows: Condition 3A (19 subjects, 48 map structures from as many distinct environments, 112 sub-maps), and condition B (54 subjects, 185 map structures from as many distinct environments, 310 sub-maps).

Condition	All features, subject-specific GDA model	A-priori features subject-specific GDA model	No subject- specific model
Condition 3A	**79.2% (RI = 0.94)**	70.8% (RI = 0.88)	41.7% (RI = 0.76)
Condition 3B	**68.6% (RI = 0.89)**	63.4% (RI = 0.83)	60.2% (RI = 0.78)

#### Discussion

The model prediction accuracies reported above are close to the estimated maximum possible prediction rates from noisy map structures (based on simulating participant distractions using random swapping), calculated at the beginning of this Section: 62.5% for condition A, and 78.0% for condition B. This shows that the model accounts well for this noisy data, despite not being able to predict 100% of subject map structures.

Even using solely features which can be objectively derived from geospatial information from participants’ specified buildings, without collecting any subjective data such as sketch maps or visual similarity judgements for the test maps, solid prediction is still possible—70.8% for condition A and 68.8% in B (although recall sequences still need to be collected in order to learn subject-specific models). This makes a subject model, once learned, applicable to any environment encountered by that subject.

These results further substantiate the plausibility of the individual psychological space and the clustering hypotheses introduced in the Introduction, and show for the first time that human spatial representation structures can be predicted in advance.

## Discussion

A growing body of evidence suggests that rather than storing spatial information within some global reference frame, human spatial memory employs local, object-centered representations [11, 12, 82, 83]. This is consistent with the much earlier proposal that spatial memories are organized according to hierarchies [6–10], as well as with recent neuronal evidence [16, 19].

In this paper, we made the first attempt to quantitatively explain and predict the local structure of spatial representations. We have found strong correlations between the probability that two buildings are co-represented in participants’ allocentric spatial representations, and features such as Euclidean distance, path distance, and visual and functional similarity. These correlations suggest that clustering based on proximity along these features is likely to give rise to the observed representation structure. We have developed multiple methods for exploring how important these features are for individual subjects (i.e. automatically learning their ‘psychological spaces’), even if only small amounts of data are available, and have developed and evaluated a predictive model of cognitive map structure based on Bayesian nonparametric clustering in these learned psychological spaces. We have shown that our model can successfully predict spatial representation structures in advance in the majority of cases.

The results from our model are very promising, but their plausibility depends on the empirical method used to expose spatial representation structure. Although the structures identified by our recall order paradigm are substantiated by their significant influence on several cognitive phenomena (Experiment 1), there is clearly room for improving the experimental methodology. After briefly outlining the implications of models of cognitive map structure, the discussion below outlines some alternative approaches, and suggests reasons for the imperfect prediction rates.

The mentioned feature correlations, as well as the predictions of the computational model and the conclusions with regard to the individual psychological space and clustering hypotheses, only concern individuals who are capable of learning structured allocentric cognitive maps. There is evidence for substantial individual differences in cognitive map formation—some participants seem to be substantially more accurate at recalling spatial relationships, or may only learn route-based representations [84–86]. This was also evident from our data—a substantial proportion of our subjects produced sketch maps which either had errors comparable to randomly generated maps, or showed no evidence of structure.

It is possible that the participants unable to produce accurate sketch maps, or not showing structure in their recall protocols, nevertheless use a clustering process to structure their representations. In principle, this would be possible even for egocentric or route-based spatial knowledge as well. However, this cannot be tested with our present paradigm. The question could be investigated in future work with a different experimental setup, for example, using error patterns in judgements of relative direction to infer representation structure (see below).

### Implications of modelling cognitive map structure

We have reported significant effects exerted by cognitive map structure on spatial memory-related performance in the Results section. Together with prior evidence on priming, map distortion, distance estimation biases, and related effects, it seems clear that representation structure is relevant to spatial memory.

Apart from psychology, its investigation is also of interest for neuroscience. Strong evidence exists for hierarchies in the neural correlates of rodent spatial memory, place cells and grid cells, specialized neuron types discovered in mammalian—and, more recently, human—brains [4, 87], and is shown to play a key role in representing space [88]. Place cells show increased activity in small, spatially localized areas, encoding spatial locations within particular spaces—with firing patterns changing significantly upon switching or changing immediate surroundings (the set of active place cells is completely different in separate environments). Grid cell firing shows a highly regular, triangular grid spanning the surface of an environment, independently of its configuration of landmarks, thus encoding a direction and distance metric.

Both of these spatially relevant neuron types have been observed to show natural hierarchies, with the granularity of representations (the sizes of the firing fields of individual cells) increasing from dorsal to ventral poles of the relevant brain areas [14, 15]. Furthermore, fragmentation in separate parts of an environment has also been observed in electrophysiological recordings of grid cells [31, 89], indicating that instead of a single ‘cognitive map’, there a manifold of sub-maps are represented in brains [16].

However, the connection between these hierarchical and/or fragmented neural representations, and cognitive representations of map structure, remains largely unexplored. The predictive modelling approach presented in this paper could facilitate and accelerate research into this connection—after a subject-specific model has been learned from a small number of environments, subjects do not need to be subjected to arduous recall sequences (or large numbers of estimations), and can quickly be tested in large numbers of virtual reality environments in an fMRI.

Models of cognitive map structure could be of interest not only to the cognitive sciences but also to neighbouring fields. For example, in geographic information science, the insight that both planning times and estimation accuracies are improved within sub-maps compared to across, together with a subject-general model (which is good enough for this purpose—see Experiment 3), could help design schematics or transit maps which are cognitively easy to use for a majority of subjects.

Furthermore, models of human spatial representation are relevant for robotics for the purpose of communicating and interacting with humans. This is a rapidly growing area, with over three million personal (non-industrial) service robots sold in 2012 (according to the World Robotics 2013 Service Robot Statistics); a figure that can be expected to grow with the increasing demands on care robotics due to the rapid ageing of the world population. A model of spatial representation structure could allow artificial agents to use and understand human-like concepts (for example, translating latitudes and longitudes to easily understandable expressions like ‘between the shopping area and the university buildings’). Approaches to conceptualize spatial representations exist only for indoor robots [90]. The present approach, in contrast, is applicable to unconstrained outdoor environments (and is demonstrated by our results to work in a human-like fashion in over a hundred cities). In the nearer future, computational models of spatial concepts could facilitate specifying vague areas (sub-maps) as goals for GPS devices.

Finally, the particular way individual subjects structure their commonly encountered environments depending on past experience and task demands could give insight into computationally more efficient spatial representations for artificial intelligence (AI). With only around 40 million principal neurons in the human Hippocampus [91], adults seem to be able to effortlessly store and recall navigation-relevant spatial details of many dozens of cities and hundreds of square kilometers. Storing a comparable amount on a trivial AI map representation such as an occupancy grid [92], with the accuracy relevant for navigation, and including rich perceptual information, is not possible using today’s hardware (let alone searching through such a vast database in split seconds, as humans are able to do). Human spatial representation structure could give inspiration for more efficient computational structures for representing space.

### Alternative empirical approaches to uncovering cognitive map structure

Since humans do not have introspective insight into their own memory structure, uncovering organization principles of spatial memory is challenging. Several methods have been proposed in the literature to investigate which reference frames, or imposed structure, might be employed by participants. Of these, the recall order paradigm was used here, and described in Materials and Methods. Its main shortcomings are the lack of robustness to outliers due to e.g. lapses of attention (mitigated by the jackknifing procedure), and the influences of phonological and morphological features of verbally cued items (mitigated by spatial cueing, as in Experiment 3). Despite these shortcomings, the structures extracted by this method have substantial influence on various cognitive phenomena, as reported in the results above.

Other experimental approaches for investigating representation structure include judgements of relative direction (JRD), in which subjects imagine standing at some specified location and heading, are asked to point to specified objects they have memorized previously. The angular error in JRD seems to be strongly affected by interobject spatial relations (rather than only depending on a global reference frame), with better accuracy for judgements aligned with the intrinsic reference frame of an array of objects both in navigation space [11, 93] and in small-scale environments in a room [94]. These experiments have utilized object arrays with clear axes of alignment, either employing a grid-like array (mainly used in small-scale experiments) or making use of single major roads or paths as intrinsic axes in large-scale surroundings. This setup limits the applicability to general environments. However, the idea of direction judgement errors induced by changes of reference frame is generalizable, and has also been used to investigate reference frames of arrays without enforced intrinsic structure [19, 83]. Because direction errors are smaller within reference frames than across [19], they could in principle be used to infer representation structure. The main disadvantage of this approach is the large number of direction estimations required to distinguish reference frames reliably, due to the large variance of direction errors. Furthermore, the number of estimations needed for pairwise comparison grows quadratically with the number of objects and / or frames (none of the cited papers compare more than two frames).

Cognitive map structure impinges on behavioural performance in several ways, most notably including biasing direction estimation (see above), distance estimation—overestimated across- and underestimated within representations [6] -, and priming, i.e. accelerated recognition latencies [8], direction estimation latencies [19], and verifications of spatial relations [20]. All of these biases in errors or response times cause the same difficulties when trying to infer the exact representation structure for a particular participant—due to large variances, a very large number of judgements is required to obtain acceptable statistical significance (and the number grows quadratically with the number of objects). How to mitigate this problem, and which of these metrics have the smallest variance and thus highest reliability for map structure extraction, as well as whether they all yield consistent structures as would be expected, remain important questions for future research on cognitive map structuring.

Assuming either no distractions, or that jackknifing can successfully eliminate the majority of outliers caused by distractions, the recall order paradigm is able to provide the most deterministic way of inferring map structure, since it does not rely on comparing distributions of errors (or response times) using significance testing. It is also deterministic over time, resulting in very similar structures to the original hierarchies when re-testing subjects several weeks later [6]. These advantages, together with the difficulty of obtaining statistically significant results from error / RT patterns with high variances, have motivated our choice for the recall order paradigm for uncovering the structures modelled in this work.

### Obstacles to predicting cognitive map structure

Our results indicate strong correlations of co-representation probability with distance, suggesting that a clustering mechanism underlies map structures, and substantiating the plausibility of our computational model. However, these conclusions are based on a number of assumptions; and it is possible that some of them might not be correct. Below, we list some possible obstacles to a predictive model based on these assumptions.

First, it might be the case that subjects did not learn allocentric spatial representations of their chosen buildings at all. They might have painstakingly constructed the sketch maps in these experiments from egocentric representations, for example by imagining egocentric vectors from a particular vantage point, and estimating distances. If subjects can accurately estimate distances, then this procedure might yield sketch maps that are better than random, despite the absence of a metric cognitive map (subjects might well do this, for example, if they have only ever visited their chosen buildings by underground public transport). However, note that 1) in this case they would be violating the experiment instructions, which state that they need to know how to walk from any of the buildings to any of the others, and 2) it is much harder to draw accurate sketch maps when estimating from only one (or few) egocentric vantage points, as opposed to when a full ‘map’ is accessible allowing the choice of any building or points between buildings as vantage points.

Second, subject cognitive maps might be unstructured. However, according to the recall order paradigm, structure is evident from the recalls of a majority of subjects and subject maps. There is also the independent evidence of several distinct local reference frames, and of local neural representations (see above).

Third, apparent structure might actually arise from non-spatial context effects or long-term memory events which happened at, or are relevant to, a sub-set of buildings or locations on a subject’s cognitive map. For example, a subject might cluster together multiple restaurants after having had dinner at all of them with her significant other. When filling out the recall sequences, she might employ her salient episodic memories of these dinners to quickly recall these restaurants (and recall them together, which would lead to the tree analysis algorithm to assume that they are clustered together). It is difficult to exclude such influences in the real-world experiments, as most buildings familiar to subjects will have some sort of episodic memories associated with them. How frequent such influences are, and to what extent they distort apparent map structure, remain questions for future research (one approach might be trying to induce meaningful episodic memories in the virtual reality experiment, and measure their effects). However, if a majority of subject map structures had been affected by such context effects (which naturally cannot be modelled with the described features), reliable prediction would not be possible at all. The observation that a majority of structures *can* be predicted suggests that these influences affect a minority of recalled structures.

Fourth, spurious structures could appear in the recall sequences from phonetic or morphological name similarity in case subjects use articulatory rehearsal to facilitate quick recall; in which case it is a natural strategy to rehearse and recall similarly sounding object names together. This was indeed a significant influence in the verbally cued experiments (Experiment 1 and 2), although much weaker than the dominating influence of spatial distance. However, it seems that the effect can be mitigated substantially by changing the cue modality from verbal to visuospatial cues, which reduces the correlations between phonetic/morphological similarity and co-representation probability to insignificant levels (see Fig 10). A further possible objection related to working memory, that the uncovered structures might be learned during the experiment (instead of arising from long-term spatial memory), can be ruled out based on the approximately uniform distribution of outlier positions (the first few sequences were not more likely to be outliers than the last few sequences, and no evidence for any learning of map structures during the real-world experiments could be found in the data, indicating that the responses were indeed indicative of long-term memory structure and not of more recent structures in working memory—see S1 File for details).

Fifth, in the real-world experiments during which subjects were not observed, they could have lightened the cognitive load and speeded up the process by either writing down the list of buildings, or sketching a map on paper, and then reading instead of recalling. Although they were explicitly instructed to do everything from memory, without looking anything up, an unfortunate side effect of the monetary re-compensation is that they have financial incentive to speed up the task (however, [95] have found no significant difference between the ratio of correct answers between Mechanical Turk participants and supervised subject from a middle-class urban neighbourhood; although there was a significant difference to student participants). The proportion of subjects ignoring task instructions can be reduced by ensuring that most of their other tasks were accepted by requesters on MTurk (in these experiments, they were required to have at least 95% approval rating on previous jobs to ensure higher data quality). Furthermore, since the easiest strategy when using a list or a sketch on paper is to always use the same ordering, this should cause recall sequences to be circular, which can be detected in the data. As would be expected, the rate of circular recalls is significantly higher for the MTurk subjects (Experiment 3)—12.6%—than for the student participants of Experiment 2—5.3%. However, they are still a minority of the data, and have been excluded in the reported analyses (as they lead to a lack of apparent structure).

**Fig 14:**
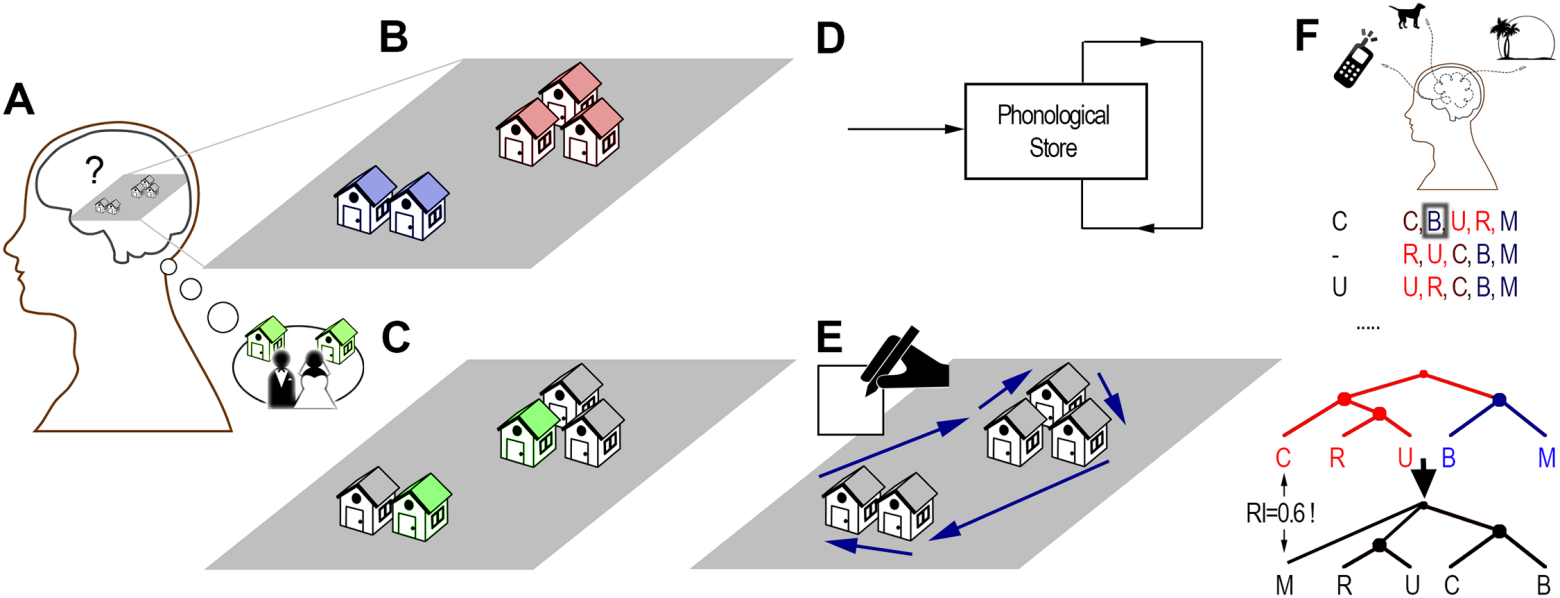
Possible obstacles to predicting subject cognitive map structures. A: Subjects may not have formed allocentric cognitive maps. B: Their maps may not have been structured. C: The apparent structure might be due to episodic memories, emotionally significant events, or other types of non-spatial long-term memory. D: Spurious structure might arise from articulatory rehearsal or other working memory strategies, instead of LTM. E: Subjects can list or sketch their buildings on paper, instead of recalling them from memory, to make the task faster and easier; usually resulting in circular recall sequences. F: Mind wandering or lapses in attention during recall sequences can cause tree analysis to reconstruct incorrect map structures.

An additional obstacle to predicting cognitive map structure is the rigidity of the tree analysis algorithm. Sub-maps are only recognized as such if they occur together, without interruption, in *every single recall sequence*. Fig 14 F illustrates an example (revisiting the example from Fig 4) where a distraction, which interrupts the sequence cued with ‘C’ and causes the participant to continue with ‘B’, for example because the distraction has reminded him of ‘B’. This causes a substantially different extracted map structure—were a well-trained predictive model to predict the correct (CRU)- (BM) sub-map structure, it would show up as an incorrect prediction, and to have a Rand index of 0.6 instead of 1.0. S1 File suggests a calculation of how many such incorrectly inferred map structures there might be in our data, based on the percentage of recognized outliers using jackknifing.

Apart from devising a less simplistic outlier detection method, one possibility to reduce the occurrence of distractions—for future work—would be timing all recall sequences, and discarding those that exceed a temporal threshold, forcing participants to re-do the recall.

## Conclusion

The way spatial memories of open, large-scale environments are structured has remained an unanswered question. In this paper, we have provided the first attempt at a quantitative answer, hypothesizing that cognitive map structure arises from clustering in some subject-specific psychological space, including (but not necessarily limited to) a list of features such as spatial distance, separating boundaries and streets, and visual and functional similarity. As this claim implies a strong dependence between whether or not objects are stored on the same representations, and these features, we have examined this dependence using subjects from over a hundred cities worldwide.

We have found that for participants who have learned structured allocentric cognitive maps, there is a strong correlation between the probability of co-representation of buildings and their distance in these features (including, perhaps surprisingly, their visual similarities). Furthermore, we report that despite the noisy inference of subject map structures, they can be predicted correctly in a majority of cases, after learning metric characterising subjects’ psychological spaces and applying a clustering method based on Bayesian models of cognition. Together, these results provide support for the individual psychological space and clustering hypotheses, and for the plausibility of a Bayesian clustering model of cognitive map structuring.

## Supporting Information

S1 File Methodological details. (PDF)

## References

[1] Tolman EC. Cognitive maps in rats and men. Psychological review. 1948;55(4):189 10.1037/h0061626 18870876

[2] O’Keefe J, Nadel L. The hippocampus as a cognitive map. vol. 3 Clarendon Press Oxford; 1978.

[3] McNaughton BL, Battaglia FP, Jensen O, Moser EI, Moser MB. Path integration and the neural basis of the’cognitive map’. Nature Reviews Neuroscience. 2006;7(8):663–78. 10.1038/nrn1932 16858394

[4] Ekstrom AD, Kahana MJ, Caplan JB, Fields TA, Isham EA, Newman EL, et al Cellular networks underlying human spatial navigation. Nature. 2003;424:184–187. 10.1038/nature01964 12968182

[5] Barry C, Lever C, Hayman R, Hartley T, Burton S, O’Keefe J, et al The boundary vector cell model of place cell firing and spatial memory. Reviews in the Neurosciences. 2006;17(1-2):71–97. 10.1515/REVNEURO.2006.17.1-2.71 16703944 PMC2677716

[6] Hirtle SC, Jonides J. Evidence of hierarchies in cognitive maps. Memory & Cognition. 1985;13(3):208–217. 10.3758/BF03197683 4046821

[7] McNamara TP. Mental representations of spatial relations. Cognitive psychology. 1986;18(1):87–121. 10.1016/0010-0285(86)90016-2 3948491

[8] McNamara TP, Hardy JK, Hirtle SC. Subjective hierarchies in spatial memory. Journal of Experimental Psychology: Learning, Memory, and Cognition. 1989;15(2):211 2522511 10.1037//0278-7393.15.2.211

[9] Holding CS. Further evidence for the hierarchical representation of spatial information. Journal of Environmental Psychology. 1994;14(2):137–147. 10.1016/S0272-4944(05)80167-7

[10] Wiener JM, Mallot HA. ’Fine-to-coarse’route planning and navigation in regionalized environments. Spatial cognition and computation. 2003;3(4):331–358. 10.1207/s15427633scc0304_5

[11] Meilinger T, Riecke BE, Bülthoff HH. Local and global reference frames for environmental spaces. The Quarterly Journal of Experimental Psychology. 2014;67(3):542–569. 10.1080/17470218.2013.821145 23972144

[12] Greenauer N, Waller D. Micro-and macroreference frames: Specifying the relations between spatial categories in memory. Journal of Experimental Psychology: Learning, Memory, and Cognition. 2010;36(4):938 20565211 10.1037/a0019647

[13] Shelton AL, McNamara TP. Systems of spatial reference in human memory. Cognitive psychology. 2001;43(4):274–310. 10.1006/cogp.2001.0758 11741344

[14] Brun VH, Solstad T, Kjelstrup KB, Fyhn M, Witter MP, Moser EI, et al Progressive increase in grid scale from dorsal to ventral medial entorhinal cortex. Hippocampus. 2008;18(12):1200–1212. 10.1002/hipo.20504 19021257

[15] Kjelstrup KB, Solstad T, Brun VH, Hafting T, Leutgeb S, Witter MP, et al Finite scale of spatial representation in the hippocampus. Science. 2008;321(5885):140–143. 10.1126/science.1157086 18599792

[16] Derdikman D, Moser EI. A manifold of spatial maps in the brain. Trends in cognitive sciences. 2010;14(12):561–569. 10.1016/j.tics.2010.09.004 20951631

[17] Gobet F, Lane PC, Croker S, Cheng PC, Jones G, Oliver I, et al Chunking mechanisms in human learning. Trends in cognitive sciences. 2001;5(6):236–243. 10.1016/S1364-6613(00)01662-4 11390294

[18] Cohen G. Hierarchical models in cognition: Do they have psychological reality? European Journal of Cognitive Psychology. 2000;12(1):1–36. 10.1080/095414400382181

[19] Han X, Becker S. One spatial map or many? Spatial coding of connected environments. Journal of Experimental Psychology: Learning, Memory, and Cognition. 2014;40(2):511 24364723 10.1037/a0035259

[20] Hommel B, Gehrke J, Knuf L. Hierarchical coding in the perception and memory of spatial layouts. Psychological Research. 2000;64(1):1–10. 10.1007/s004260000032 11109863

[21] Reitman JS, Rueter HH. Organization revealed by recall orders and confirmed by pauses. Cognitive Psychology. 1980;12(4):554–581. 10.1016/0010-0285(80)90020-1 7418369

[22] Cooke NM, Durso FT, Schvaneveldt RW. Recall and measures of memory organization. Journal of Experimental Psychology: Learning, Memory, and Cognition. 1986;12(4):538.

[23] Muller RU, Stead M, Pach J. The hippocampus as a cognitive graph. The Journal of general physiology. 1996;107(6):663–694. 10.1085/jgp.107.6.663 8783070 PMC2219396

[24] Gillner S, Mallot HA. Navigation and acquisition of spatial knowledge in a virtual maze. Journal of Cognitive Neuroscience. 1998;10(4):445–463. 10.1162/089892998562861 9712675

[25] Trullier O, Meyer JA. Animat navigation using a cognitive graph. Biological Cybernetics. 2000;83(3):271–285. 10.1007/s004220000170 11007301

[26] Sato N, Yamaguchi Y. Online formation of a hierarchical cognitive map for object-place association by theta phase coding. Hippocampus. 2005;15(7):963 10.1002/hipo.20110 16145691

[27] Sato N, Yamaguchi Y. Spatial-area selective retrieval of multiple object–place associations in a hierarchical cognitive map formed by theta phase coding. Cognitive neurodynamics. 2009;3(2):131–140. 10.1007/s11571-008-9074-9 19130301 PMC2678200

[28] Hirtle SC. Representational structures for cognitive space: Trees, ordered trees and semi-lattices In: Spatial Information Theory a Theoretical Basis for GIS. Springer; 1995 p. 327–340.

[29] Kavouras M, Kokla M. A method for the formalization and integration of geographical categorizations. International Journal of Geographical Information Science. 2002;16(5):439–453. 10.1080/13658810210129120

[30] Meilinger T. The network of reference frames theory: A synthesis of graphs and cognitive maps In: Spatial Cognition VI. Learning, Reasoning, and Talking about Space. Springer; 2008 p. 344–360.

[31] Derdikman D, Whitlock JR, Tsao A, Fyhn M, Hafting T, Moser MB, et al Fragmentation of grid cell maps in a multicompartment environment. Nature neuroscience. 2009;12(10):1325–1332. 10.1038/nn.2396 19749749

[32] Thomas R, Donikian S. A spatial cognitive map and a human-like memory model dedicated to pedestrian navigation in virtual urban environments In: Spatial Cognition V Reasoning, Action, Interaction. Springer; 2007 p. 421–438.

[33] Raubal M, Egenhofer MJ, Pfoser D, Tryfona N. Structuring space with image schemata: Wayfinding in airports as a case study In: Spatial Information Theory A Theoretical Basis for GIS. Springer; 1997 p. 85–102.

[34] Reineking T, Kohlhagen C, Zetzsche C. Efficient wayfinding in hierarchically regionalized spatial environments In: Spatial Cognition VI. Learning, Reasoning, and Talking about Space. Springer; 2008 p. 56–70.

[35] Byrne P, Becker S, Burgess N. Remembering the past and imagining the future: a neural model of spatial memory and imagery. Psychological review. 2007;114(2):340 10.1037/0033-295X.114.2.340 17500630 PMC2678675

[36] Voicu H. Hierarchical cognitive maps. Neural Networks. 2003;16(5):569–576. 10.1016/S0893-6080(03)00095-9 12850009

[37] Wang RF, Spelke ES. Human spatial representation: Insights from animals. Trends in cognitive sciences. 2002;6(9):376–382. 10.1016/S1364-6613(02)01961-7 12200179

[38] Hurts K. Spatial memory as a function of action-based and perception-based similarity In: Proceedings of the Human Factors and Ergonomics Society Annual Meeting. vol. 52 SAGE Publications; 2008 p. 1165–1169.

[39] Madl T, Chen K, Montaldi D, Trappl R. Computational cognitive models of spatial memory in navigation space: A review. Neural Networks. 2015;65:18–43. 10.1016/j.neunet.2015.01.002 25659941

[40] Kriegeskorte N, Kievit RA. Representational geometry: integrating cognition, computation, and the brain. Trends in cognitive sciences. 2013;17(8):401–412. 10.1016/j.tics.2013.06.007 23876494 PMC3730178

[41] Shepard RN. Toward a universal law of generalization for psychological science. Science. 1987;237(4820):1317–1323. 10.1126/science.3629243 3629243

[42] Edelman S. Representation is representation of similarities. Behavioral and Brain Sciences. 1998;21(04):449–467. 10.1017/S0140525X98001253 10097019

[43] Gärdenfors P. Conceptual spaces: The geometry of thought. MIT press; 2004.

[44] Kriegeskorte N, Mur M, Bandettini P. Representational similarity analysis–connecting the branches of systems neuroscience. Frontiers in systems neuroscience. 2008;2 10.3389/neuro.06.004.2008 19104670 PMC2605405

[45] Collin SH, Milivojevic B, Doeller CF. Memory hierarchies map onto the hippocampal long axis in humans. Nature Neuroscience. 2015;18(11):1562–1564. 10.1038/nn.4138 26479587 PMC4665212

[46] Naveh-Benjamin M, McKeachie WJ, Lin YG, Tucker DG. Inferring students’ cognitive structures and their development using the “ordered tree technique”. Journal of Educational Psychology. 1986;78(2):130 10.1037/0022-0663.78.2.130

[47] Crump MJ, McDonnell JV, Gureckis TM. Evaluating Amazon’s Mechanical Turk as a tool for experimental behavioral research. PloS one. 2013;8(3):e57410 10.1371/journal.pone.0057410 23516406 PMC3596391

[48] Gower JC. Generalized procrustes analysis. Psychometrika. 1975;40(1):33–51. 10.1007/BF02291478

[49] MacKinnon JG. Bootstrap hypothesis testing Handbook of Computational Econometrics. 2009; p. 183–213.

[50] Shepard RN, Chipman S. Second-order isomorphism of internal representations: Shapes of states. Cognitive psychology. 1970;1(1):1–17. 10.1016/0010-0285(70)90002-2

[51] Choe Y. Second order isomorphism: A reinterpretation and its implications in brain and cognitive sciences. In: Proceedings of the 24th Annual Confernce of the Cognitive Science Society. Lawrence Erlbaum Associates; 2002. p. 190–195.

[52] DiCarlo JJ, Cox DD. Untangling invariant object recognition. Trends in cognitive sciences. 2007;11(8):333–341. 10.1016/j.tics.2007.06.010 17631409

[53] Shepard RN. Stimulus and response generalization: A stochastic model relating generalization to distance in psychological space. Psychometrika. 1957;22(4):325–345. 10.1007/BF02288967 13563763

[54] Gablonsky JM, Kelley CT. A locally-biased form of the DIRECT algorithm. Journal of Global Optimization. 2001;21(1):27–37. 10.1023/A:1017930332101

[55] Jones DR, Perttunen CD, Stuckman BE. Lipschitzian optimization without the Lipschitz constant. Journal of Optimization Theory and Applications. 1993;79(1):157–181. 10.1007/BF00941892

[56] Rand WM. Objective criteria for the evaluation of clustering methods. Journal of the American Statistical association. 1971;66(336):846–850. 10.1080/01621459.1971.10482356

[57] Settles B. Active learning literature survey. Computer Sciences Technical Report 1648. 2010;.

[58] Hosmer DW, Lemeshow S. Applied logistic regression. John Wiley & Sons; 2004.

[59] Lewis DD, Gale WA. A sequential algorithm for training text classifiers In: Proceedings of the 17th annual international ACM SIGIR conference on Research and development in information retrieval. Springer-Verlag New York, Inc.; 1994 p. 3–12.

[60] Yang L, Jin R. Distance metric learning: A comprehensive survey. Michigan State Universiy 2006;2.

[61] Baghshah MS, Shouraki SB. Kernel-based metric learning for semi-supervised clustering. Neurocomputing. 2010;73(7):1352–1361. 10.1016/j.neucom.2009.12.009

[62] Chitta R, Jin R, Havens TC, Jain AK. Approximate kernel k-means: Solution to large scale kernel clustering In: Proceedings of the 17th ACM SIGKDD international conference on Knowledge discovery and data mining. ACM; 2011 p. 895–903.

[63] Ong CS, Williamson RC, Smola AJ. Learning the kernel with hyperkernels In: Journal of Machine Learning Research; 2005 p. 1043–1071.

[64] Bensmail H, Celeux G. Regularized Gaussian discriminant analysis through eigenvalue decomposition. Journal of the American statistical Association. 1996;91(436):1743–1748. 10.1080/01621459.1996.10476746

[65] Gershman SJ, Blei DM. A tutorial on Bayesian nonparametric models. Journal of Mathematical Psychology. 2012;56(1):1–12. 10.1016/j.jmp.2011.08.004

[66] Sanborn AN, Griffiths TL, Navarro DJ. A more rational model of categorization. In: Proceedings of the 28th annual conference of the cognitive science society; 2006. p. 726–731.

[67] Griffiths TL, Canini KR, Sanborn AN, Navarro DJ. Unifying rational models of categorization via the hierarchical Dirichlet process. In: Proceedings of the 29th annual conference of the cognitive science society; 2007. p. 323–328.

[68] Tenenbaum JB, Kemp C, Griffiths TL, Goodman ND. How to grow a mind: Statistics, structure, and abstraction. science. 2011;331(6022):1279–1285. 10.1126/science.1192788 21393536

[69] Canini KR, Shashkov MM, Griffiths TL. Modeling Transfer Learning in Human Categorization with the Hierarchical Dirichlet Process In: ICML; 2010 p. 151–158.

[70] Gibson BR, Rogers TT, Zhu X. Human Semi-Supervised Learning. Topics in cognitive science. 2013;5(1):132–172. 10.1111/tops.12010 23335577

[71] Blei DM, Jordan MI, et al Variational inference for Dirichlet process mixtures. Bayesian analysis. 2006;1(1):121–143. 10.1214/06-BA104

[72] Hughes MC, Sudderth E. Memoized online variational inference for Dirichlet process mixture models In: Advances in Neural Information Processing Systems; 2013 p. 1133–1141.

[73] Nachar N. The Mann-Whitney U: a test for assessing whether two independent samples come from the same distribution. Tutorials in Quantitative Methods for Psychology. 2008;4(1):13–20.

[74] Nosofsky RM. Attention, similarity, and the identification–categorization relationship. Journal of experimental psychology: General. 1986;115(1):39 10.1037/0096-3445.115.1.39 2937873

[75] Howard LR, Javadi AH, Yu Y, Mill RD, Morrison LC, Knight R, et al The hippocampus and entorhinal cortex encode the path and Euclidean distances to goals during navigation. Current Biology. 2014;24(12):1331–1340. 10.1016/j.cub.2014.05.001 24909328 PMC4062938

[76] Ekstrom AD. Why vision is important to how we navigate. Hippocampus 2015;. 10.1002/hipo.22449 PMC4449293 25800632

[77] Barsalou LW. Grounded cognition. Annu Rev Psychol. 2008;59:617–645. 10.1146/annurev.psych.59.103006.093639 17705682

[78] Philips L. The double metaphone search algorithm. C/C++ users journal. 2000;18(6):38–43.

[79] Khorsi A. On morphological relatedness. Natural Language Engineering. 2013;19(04):537–555. 10.1017/S1351324912000071

[80] Kraemer HC. Biserial correlation Encyclopedia of statistical sciences. 1982;.

[81] Redner RA, Walker HF. Mixture densities, maximum likelihood and the EM algorithm. SIAM review. 1984;26(2):195–239. 10.1137/1026034

[82] Marchette SA, Shelton AL. Object properties and frame of reference in spatial memory representations. Spatial Cognition & Computation. 2010;10(1):1–27. 10.1080/13875860903509406

[83] Chen X, McNamara T. Object-centered reference systems and human spatial memory. Psychonomic bulletin & review. 2011;18(5):985–991. 10.3758/s13423-011-0134-5 21786070

[84] Blajenkova O, Motes M, Kozhevnikov M. Individual differences in the representations of novel environments. Journal of Experimental Psychology. 2005;25:97–109.

[85] Ishikawa T, Montello DR. Spatial knowledge acquisition from direct experience in the environment: Individual differences in the development of metric knowledge and the integration of separately learned places. Cognitive Psychology. 2006;52:93–129. 10.1016/j.cogpsych.2005.08.003 16375882

[86] Weisberg SM, Schinazi VR, Newcombe NS, Shipley TF, Epstein RA. Variations in cognitive maps: Understanding individual differences in navigation. Journal of Experimental Psychology: Learning, Memory and Cognition. 2013;. 10.1037/a0035261 24364725

[87] Jacobs J, Weidemann CT, Miller JF, Solway A, Burke JF, Wei XX, et al Direct recordings of grid-like neuronal activity in human spatial navigation. Nature Neuroscience. 2013;16(9):1188–1190. 10.1038/nn.3466 23912946 PMC3767317

[88] Moser EI, Kropff E, Moser MB. Place cells, grid cells, and the brain’s spatial representation system. Annual review of neuroscience. 2008;31:69–89. 10.1146/annurev.neuro.31.061307.090723 18284371

[89] Frank LM, Brown EN, Wilson M. Trajectory encoding in the hippocampus and entorhinal cortex. Neuron. 2000;27(1):169–178. 10.1016/S0896-6273(00)00018-0 10939340

[90] Zender H, Mozos OM, Jensfelt P, Kruijff GJ, Burgard W. Conceptual spatial representations for indoor mobile robots. Robotics and Autonomous Systems. 2008;56(6):493–502. 10.1016/j.robot.2008.03.007

[91] Andersen P, Morris R, Amaral D, Bliss T, O’Keefe J. The hippocampus book. Oxford University Press; 2006.

[92] Elfes A. Using occupancy grids for mobile robot perception and navigation. Computer. 1989;22(6):46–57. 10.1109/2.30720

[93] McNamara TP, Rump B, Werner S. Egocentric and geocentric frames of reference in memory of large-scale space. Psychonomic Bulletin & Review. 2003;10(3):589–595. 10.3758/BF03196519 14620351

[94] Mou W, McNamara TP. Intrinsic frames of reference in spatial memory. Journal of Experimental Psychology: Learning, Memory, and Cognition. 2002;28(1):162 11827078 10.1037/0278-7393.28.1.162

[95] Goodman JK, Cryder CE, Cheema A. Data collection in a flat world: The strengths and weaknesses of Mechanical Turk samples. Journal of Behavioral Decision Making. 2013;26(3):213–224. 10.1002/bdm.1753

