# Supplementary material for: Exploring the Structure of Spatial Representations
Source: PLoS One. 2016 Jun 27;11(6):e0157343. doi: 10.1371/journal.pone.0157343 (PMC4922593; doi:10.1371/journal.pone.0157343)
Supplement: S1 File — (PDF) [file pone.0157343.s001.pdf]

Exploring the structure of spatial representations - Supplementary Information

Tamas Madl[a,b,*], Stan Franklin[c], Ke Chen[a], Robert Trappl[b], Daniela Montaldi[d]

[a]*School of Computer Science, University of Manchester, Manchester M13 9PL, UK*
[b]*Austrian Research Institute for Artificial Intelligence, Vienna A-1010, Austria*
[c]*Institute for Intelligent Systems, University of Memphis, Memphis TN 38152, USA*
[d]*School of Psychological Sciences, University of Manchester, Manchester M13 9PL, UK*

1. Tree analysis algorithm

We used an algorithm to extract map structure from recall orders which is functionally equivalent to the ordered tree algorithm used in prior work (Hirtle & Jonides, 1985; McNamara, 1986; McNamara et al., 1989), with the exception that we disregard order information (whether or not the leaves were always recalled in a particular ordering). The algorithm takes a list of recall protocols, as well as cues, and all possible buildings, and returns the map structure (all sets of buildings which always occur together).

Algorithm 1.1: EXTRACTMAPSTRUCTURE(*Protocols*, *Cues*, *Buildings*)

$submaps \leftarrow \{\}$
for each $tuplelength \in (1, |Buildings| - 1)$
    for each $C \in Combinations(Buildings, tuplelength)$
        $occurseverywhere \leftarrow True$
        for each $p \in (0, |Protocols|)$
            $perm \leftarrow Permutations(C)$
            if $Cues[p] \notin C$ **and** $\forall(PC \in perm : PC \notin Protocols[p])$
                $occurseverywhere \leftarrow False$
                break
        if $occurseverywhere$
            $submaps \leftarrow submaps \cup C$

*tamas.madl@gmail.com

The algorithm iterates through all possible tuple lengths, and generates all possible combinations at the current tuple length. For these combinations C, it checks whether any permutation of C occurs uninterrupted in all protocols (i.e. whether all buildings in C have been recalled together); if so, C is added to the list of submaps. Notably, this check is only performed if C is not cued (line 7). It was argued in previous literature (Hirtle & Jonides, 1985; McNamara et al., 1989) that cueing can disrupt the re-call process. Therefore containment in all protocols is only tested for combinations which do not contain a cue, in order to avoid erroneously disregarding sub-maps which consistently occur together in all recall protocols except in those in which the cue has disrupted the natural recall order.

1.1. Reliability of the ordered tree paradigm

There are theoretical as well as practical limits on the predictability of cognitive map structures. The Section 'Obstacles to predicting cognitive map structure' in the main text discusses these in more detail and suggests some solutions. Here, we shall focus on the main issue concerning data analysis, namely detecting and removing outliers caused by distractions or lapses of attention. If a set of buildings that are actually co-represented on a sub-map in a subjects' spatial memory is recalled together most of the time, but the subject is distracted during one of the recall sequences, and recalls a different (not co-represented) building instead, the subsequently extracted structure will be incorrect (since tree analysis requires items to occur together in *every* recall sequence in order to identify a sub-map). Even a single distraction during the 7 or 10 (in Experiment 3 A or B) recall sequences per trial can yield substantially different structures (see example in Figure 14 in the main text, in which a distraction causes a drop of 0.6 in the Rand index to the correct structure).

The jackknifing procedure we use to eliminate outliers was suggested by the authors pioneering the recall order paradigm (Hirtle & Jonides, 1985; McNamara et al., 1989) to mitigate this issue, but relies on statistical significance testing to identify those outliers, and thus occasionally fails to do so due to the small number of recall sequences collected in our experiments (a necessary limit arising from the need to collect multiple different map structures for training and testing a predictive model — subjects already took up to 3.5 hours for these experiments even with this small number of sequences).

It is possible to estimate the effectiveness of jackknifing in our data — and the percentage of incorrectly inferred and thus unpredictable map structures

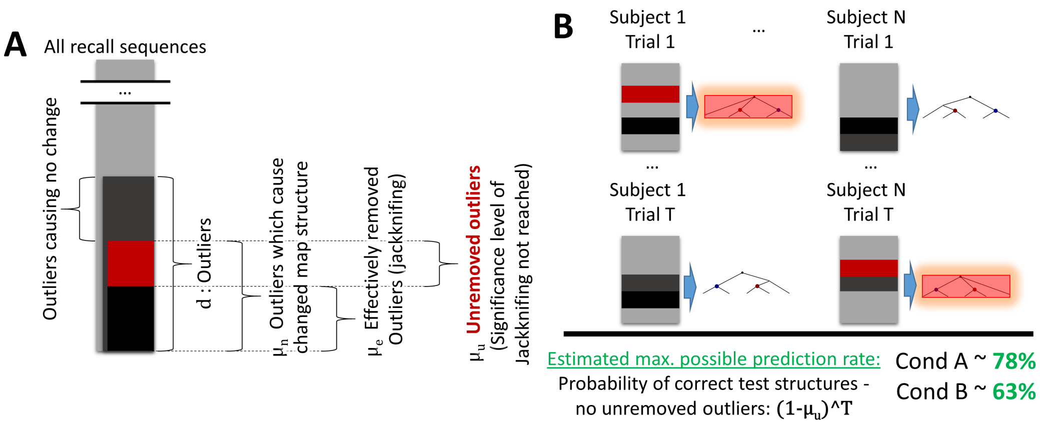

Figure 1: Estimated maximum possible prediction rate using the data in Experiment 3. A: Assuming that distractions / lapses of attention manifest as randomly swapped items in recall sequences (and cause changes in the inferred tree structures), a substantial number of them cannot be detected using the outlier detection procedure (jackknifing) proposed in the seminal work on hierarchical cognitive maps and employed in this paper. B: Undetected outliers in recall sequences cause a number of inferred map structures to be incorrect. This results in a percentage of map structures not predictable even by good models.

resulting from it (see Figure 1). To do this, we simulated distractions by randomly swapping two items in one of the sequences in each trial. This is a reasonable model of distractions, since the only way subjects can make mistakes is by changing the order of their input (they are forced to repeat the trial if they omit or incorrectly recall an item).

The *number* of simulated distractions (frequency of swapped items) makes no difference to the estimated *percentage* of outliers that are not caught and excluded by jackknifing. We used one distraction per trial (however, the following results stayed the same with 0.5 or 2 distractions per trial). For the 5 buildings maps (and 7 recall sequences), and averaging over 100 runs, each with a single random non-cue lapse for all subjects, simulated distractions cause changes in map structure (relevant outliers) in $\mu_n = 65.4\%$, $\sigma_n = 3.7\%$, and within these, outlier removal is effective in $\mu_e = 59.4\%$, $\sigma_e = 5.0\%$. The situation is somewhat better on the 8 building maps, due to the larger numbers of sequences collected and thus higher statistical power — here, outlier removal is effective in $\mu_e = 56.0\%$, $\sigma_e = 8.1\%$ of the cases (and necessary only in $\mu_n = 33.2\%$, $\sigma_n = 6.0\%$). This leaves on average $\mu_u = 26.6\%$ ($\sigma_u = \sqrt{\sigma_e^2 * \sigma_n^2 + \mu_e^2 * \sigma_n^2 + \mu_n^2 * \sigma_e^2} = 3.9\%$) of disruptive simulated

lapses of attention for condition A, and $\mu_u = 15.0\%$ ($\sigma_u = 4.3\%$) for condition B, which cannot be mitigated by jackknifing.

If we assume this uniform random swapping to be a reasonable approximation of subject distractions, this would mean that apart from the approximately $o = 9.5\%$ of sequences which were successfully removed as outliers using jackknifing (and thus part of the effective 59.4% or 56% for cond. A and B), there would be an expected additional $\mu_o = 6.5\%$ ($\sigma_o = 0.1\%$) of sequences for condition A, and expected $\mu_o = 2.5\%$ ($\sigma_o = 0.7\%$) for condition B, which would likely be outliers causing structure changes which have not been removed by jackknifing because of the lack of statistical significance. It follows that the expected probability of extracting correct map structures under these assumptions — and thus the maximum possible prediction rate — is around $(1-0.065)^7 \simeq 63\%$ for condition A (since there are 7 sequences per trial), and around $(1-0.025)^{10} \simeq 78\%$ for condition B (since there are 10 sequences per trial).

To summarize, the observation that not all simulated distractions (outliers) can be identified and omitted by the jackknifing procedure strongly suggests that the data collected from human subjects also contains outliers not caught by jackknifing. Thus, these outliers prevent perfect prediction of subject map structures. Figure 1 summarizes this reasoning and the maximum possible prediction rates estimated based on it for both conditions.

2. Full list of cities chosen by included subjects

The map in Figure 2 provides a visual overview over all cities within which spatial memory data has been collected from the participants.

List of cities in Experiment 1: Albany, Albuquerque, Ames, Ann Arbor, Austin, Baltimore, Belgrade, Belmopan, Buffalo, Chennai, Chicago, Chico, Cincinnati, Corvallis, Cupertino, Denton, Denver, Dunmore, Fort Collins, Gobichettipalayam, Hampton, Klamath Falls, Kochi, Lakeland, Las Vegas, Los Angeles, Madurai, Miami, Minneapolis, Miramar, Mount Kisco, New York, Orange (FL), Pittsburgh, Pittsfield Charter Township, Potterville, Reno, Rome, San Angelo, San Bernardino, San Diego, Somerville, Springfield, Strasbourg, White Salmon, Williamsburg, Wilmington.

List of cities in Experiment 3A: Alameda, Austin, Beacon, Bedford, Belleville, Bellingham, Bengaluru, Berkeley, Bloomington, Boston, Bowie, Bowling Green (KY), Brooksville, Brownsville, Buffalo, Burlington, Cambridge, Camden, Cape Girardeau, Castlerock, Chicago, Cincinnati, College Station (TX), Colombo, Columbia, Denver, Desert Hot Springs (CA), Desoto, Duluth, Eastbrunswick, Edinburgh, Fairway, Farmersville, Fayetteville, Franklin, Germantown, Gettysburg, Glasgow, Goleta, Harwoodheights, Hemet, Highridge, Hollywood, Holt, Houston, Islavista, Jaipur, Karur, Keller, Lackawanna, Lake

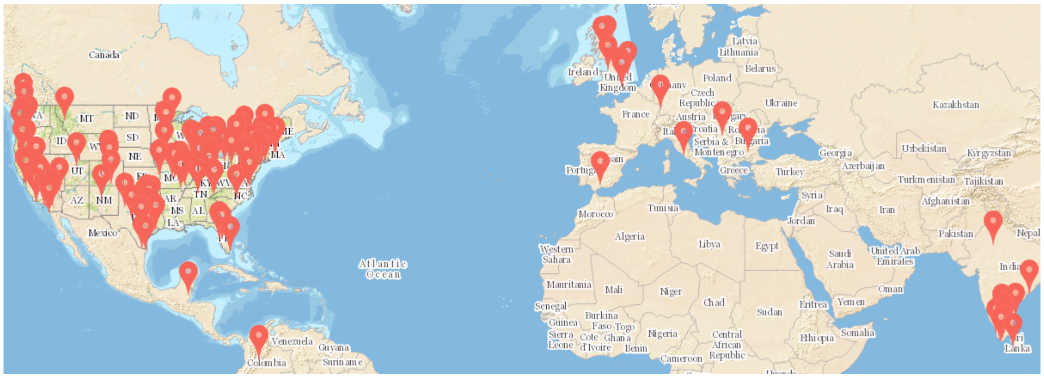

Figure 2: Overview over the 149 cities chosen by subjects in Experiments 1, 3A and 3B.

Oswego, Land O' Lakes, Lindsay, Little River-Academy (TX), Live Oak (TX), London, Lubbock, Marthandam, Mayfield, Minneapolis, Mission, Nagercoil, New York, Norridge, Orange, Overland Park (KA), Owensville, Palmsprings, Perryville, Pigeonforge, Poplarbluff, Portland, Poughkeepsie, Princeton, Provo, Revere, Rochester, Rochester Hills, Roeland Park, Salem, San Antonio, San Diego, Sanger, Savage, South Bend, Southport, Springboro, Springhill, St. Charles, Stony Brook (NY), St. Peters, Temple, Tirunelveli, Towson, Visakhapatnam, Warren, Weatherford, Wilmington, Xenia, Ypsilanti.

List of cities in Experiment 3B: Algonquin, Ashland, Chicago, Columbia, Jefferson City, Kansas City, Knoxville, Lexington, Linden, Medford, Minneapolis, Missoula, Mound, Overland Park, Portland, Seattle, Stara Zagora.

3. Exclusion of learning effects

A possible criticism of our results could be the claim that the structure apparent from the recall protocol orderings is being learned by the subjects during the recall process, as opposed to being an inherent property of their long-term memory (LTM). Our analysis procedure assumes one consistent structure in LTM underlying the recall protocols; and excludes possible 'outliers' using the jackknifing procedure (i.e. protocols which, when included, would statistically significantly change the resulting structure, are excluded from analysis).

If this assumption was incorrect, and subjects learned the structure during the experiment - or, alternatively, re-learned a different structure, then this would be apparent from the pattern of omitted recall protocols. Specifically, it would mean a significantly larger number of omitted early protocols compared to late protocols (the first, or first few protocols would be inconsistent with the learned structure more often than the last few).

**Exp. 1**	**Exp. 2**	**Exp. 3A**	**Exp. 3B**
$p = 0.886 > 0.01$	$p = 0.015 > 0.01$	$p = 0.146 > 0.01$	$p = 0.495 > 0.01$
$c = 2.339$	$c = 15.698$	$c = 9.538$	$c = 8.393$

Table 1: Results of chi-squared tests against the null hypothesis that there is no learning effect in the recall protocol data, i.e. that early recall protocols are as likely to be outliers than late recall protocols (p is the p-value of the test; c denotes the chi square test statistic). The non-significance of the results suggests that our recall order paradigm measures a property of long-term memory, and not something learned during the recall trials.

To test whether this learning effect can be observed, we have tested the distributions of omitted recall protocols against the null hypothesis that the likelihood of omissions was uniform (just as likely to occur for the first few as for the last few protocols), using a chi-square test. The table below shows the results.

For the real-world experiments, the null hypothesis cannot be rejected; thus, it is likely that there is no learning effect, and that our recall order paradigm indeed measures structures which have already been committed to LTM before the experiment. For the virtual reality experiment (Exp. 2), there seems to be some small non-uniformity, although not significant at $\alpha = 0.01$. However, contrary to the objection that the structure arises from learning during the recall trials, early protocols were less likely [1], instead of more likely, to be excluded as outliers compared to late protocols.

4. Separability of co-represented and not co-represented building pairs

The co-representation correlations reported in Section 3.3 of the main text raise hopes of straightforward predictability - what if a simple distance thresholding or linear decision boundary in the reported feature space is capable of fully explaining cognitive map structure, even for the random testing environments? Unfortunately, within sub-map and across sub-map building pairs are not linearly separable; and difficult to separate in general, even with complex state of the art classifiers.

[1]The frequency of omissions in Experiment 2 were: 1.1% for the protocols presented first, 0.7% for those at position 2, 1.3% at position 3, 1.6% at position 4, 1.7% at position 5, 1.9% at position 6, and 1.8% at position 7

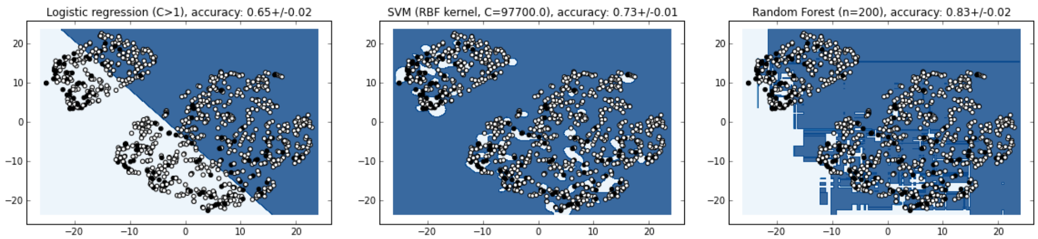

Figure 3: Pairs of buildings in the space of all features, and their separability according to co-representation, in Experiment 2 using three different classifiers: logistic regression (left), Support Vector Machine with RBF-kernel (middle) and Random Forest (right). Each point represents a building pair (filled black if both buildings lie on the same sub-map, and white if they do not), with its position being a two-dimensional projection of the full six-dimensional feature space using t-SNE. Although 2D decision boundaries are visualized, the reported classifier accuracies were obtained in the original feature space, using 10-fold cross validation and after hyperparameter optimization.

Figure 3 shows the distances of all pairs of buildings in all features in Experiment 2 , normalized by dividing each feature by its standard deviation for each participant map, and compressed down to two-dimensional space for visualization using t-SNE (Van der Maaten & Hinton, 2008) (without normalization across buildings of each map, classifiers are unable to perform above chance). Apart from the building pairs (which concentrate into two groups according to function - shops and houses), decision boundaries obtained with three different classifiers are also plotted. Although there is a trend of building pairs being more likely to be on the same sub-map when closer together (higher concentration of same-map pairs towards the lower left), the data is clearly not well-separable. As can be seen from this Figure, accurate prediction of full subject map structures - or even whether single building pairs belong to the same sub-map - using simple classification is impossible using naive approaches. More complex machine learning algorithms such as random forests (state-of-the art classifiers based on ensembles of decision trees) (Breiman, 2001) can predict for around 83% of building pairs whether they belong to the same representation (note that the accuracies were obtained by classifying the full high-dimensional data set, not just the 2D projection plotted in Figures 3 and 4). However, the map structures collected in our experiments contain 10 and 28 pairs (in the 5-building and 8-building maps), which would make the probability of full map structures - all pairs - being predicted correctly using this approach 15.5% and 0.5% respectively (the situation is even worse in real-world environments, as can

be seen in the next section).

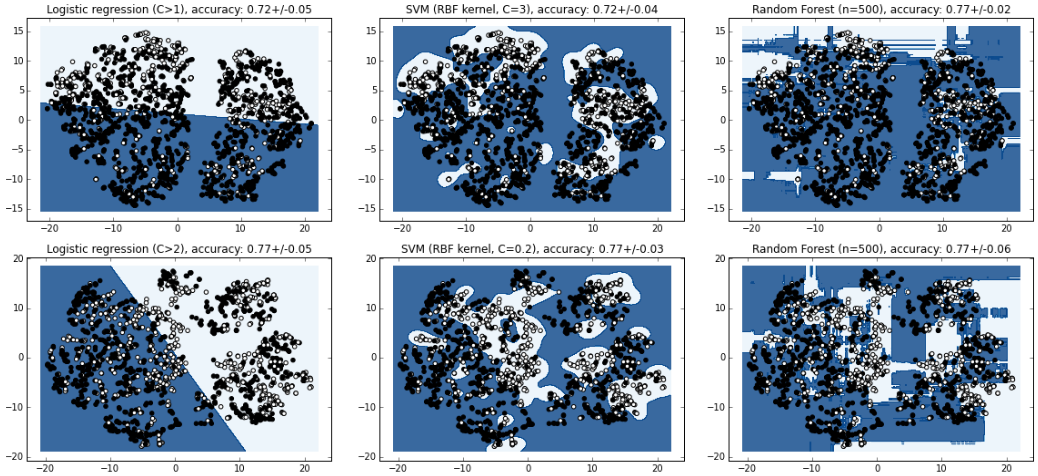

Figure 4: Pairs of buildings in the space of all features, and their separability according to co-representation, in Experiment 3 using three different classifiers: logistic regression (left), Support Vector Machine with RBF-kernel (middle) and Random Forest (right). Top: Condition A. Bottom: Condition B

In the more complex real-world setting of Experiment 3 , separating pairs of buildings which belong to the same sub-map and those belonging to different sub-maps is even more difficult than in virtual reality environments, as shown by Figure 4 and evidenced by the lower accuracies obtained after 10-fold cross validation. This Figure shows the distances of all pairs of buildings in all features, normalized by dividing each feature by its standard deviation for each participant map, and compressed down to two-dimensional space for visualization using t-SNE (Van der Maaten & Hinton, 2008). Note that the Figure shows prediction accuracies of pairs of buildings (whether or not a pair was represented on the same sub-map), and not of entire map structures. To correctly predict a map structure, all pairs within would need to be predicted correctly. Given the 77% accuracy of the best classifier in Figure 4, correct predictions based on classification are even more unlikely than in Experiment 2 ($0.77^{\binom{5}{2}} = 7.3\%$ in condition A, and $0.77^{\binom{8}{2}} = 0.0\%$ in condition B).

5. The GDA+DP-GMM Model

In order to learn a suitable metric for our data, we had to develop a novel metric learning method, since the assumptions made by existing methods do not hold in our case. Neither the linear separability assumption (made by linear metric learning), nor the prerequisite of roughly isotropic variances along the features (made by RBF-based methods (Ong et al., 2005)) is the case for all subjects in our dataset. Furthermore, a GDA-based metric can naturally incorporate the hypothesis that same sub-map building pair differences should be located close to the origin, and should be separable from different-map building pairs (these two distributions of pair differences can be naturally modelled using Gaussian distributions) - see Figure 5 in the main text.

Our proposed method can be seen as a novel approach to perform non-linear metric learning using weak supervision in the form of pairwise constraints, in order to improve clustering performance, as pioneered by Xing et al. (2002). The problem to be solved can be defined as follows. Let $\mathcal{X} = (\boldsymbol{x}_i, ..., \boldsymbol{x}_n)$ be the feature vector representation of n objects which are to be clustered, where $x_i \in \mathbb{R}^D$ are vectors with D dimensions. Let the set of m given labelled pairwise co-representation constraints be denoted by $\mathcal{C}$, where $|\mathcal{C}| = m$, and $c_{i,j} \in \mathcal{C}$ is

$$c_{i,j} = \begin{cases} 1, & \text{if } i \text{ and } j \text{ belong to the same sub-map (co-represented)} \\ 0, & \text{if } i \text{ and } j \text{ belong to different sub-maps (not co-represented)} \end{cases} \quad (1)$$

Our ultimate goal is to group the n objects into K clusters (‘sub-maps’), such that objects of the same cluster are more similar to each other than to those of different clusters; taking into account the provided pairwise constraints to learn a good similarity metric for the given data.

Conventional approaches leveraging non-linear metric learning for this problem try to find a kernel Φ such that the clustering resulting from using the distance metric defined by that kernel, $d^2(x_1, x_2) = (\Phi(x_1) - \Phi(x_2))^\intercal(\Phi(x_1) - \Phi(x_2))$, does not violate the provided constraints (ensures co-represented pairs are closer than other pairs, if possible), and often employ RBF kernels for this purpose, e.g. Baghshah & Shouraki (2010); Chitta et al. (2011).

In contrast, the proposed framework aims to learn the distribution of co-representation probabilities (whether or not two object should be linked)

from the provided set of constraints, and constructs a pseudo-metric based on a generative model of co-representation probabilities. Crucially, this probabilistic model is defined on the vector space of absolute pairwise differences (APD), which allows learning the importance of each feature (a challenge for RBF kernels for data with non-isotropic variance). The metric based on this generative model is a pseudo-metric, because it does not satisfy the conditions of subadditivity, $d(x,z) \leq d(x,y) + d(y,z)$ and the identity of discernibles, $d(x,y) = 0$ if and only if $x = y$.

Let $[\Delta \boldsymbol{x_{i,j}}]_+ = \left(|\boldsymbol{x_{i,k}} - \boldsymbol{x_{j,k}}|\right)_{k=1}^{m}$ be the representation of each pair of objects (i,j) in APD vector space. The co-representation probability distribution, i.e. the posterior probability of any pair of objects belonging to the same cluster, given a pair of objects and some model parameters $\boldsymbol{\theta}$ is then

$$p(c = 1|\Delta \boldsymbol{x}, \boldsymbol{\theta}) \propto p(\Delta \boldsymbol{x}|c = 1, \boldsymbol{\theta}) p(c = 1|\boldsymbol{\theta}) \tag{2}$$

The likelihood $p(c = 1|\Delta \boldsymbol{x}, \boldsymbol{\theta})$, the model parameters $\boldsymbol{\theta}$ (as well as the prior) can be estimated from $\mathcal{X}$ and $\mathcal{C}$, even in closed form, using Gaussian Discriminant Analysis (GDA). This yields a suitable non-linear pseudo-metric based on this probability distribution - see Equation (3) -, such that objects likely to belong to the same cluster will be close, and those likely to belong to different clusters will be far apart; with these distances directly depending on co-representation probabilities.

$$d_m(\boldsymbol{x}_1, \boldsymbol{x}_2; \boldsymbol{\theta}) = 1 - p(c = 1|\Delta \boldsymbol{x}, \boldsymbol{\theta}) = p(c = 0|\Delta \boldsymbol{x}, \boldsymbol{\theta}) \tag{3}$$

A metric is well-suited for clustering if within-cluster instances are closer than across-cluster instances according to it; i.e. if for any $\Delta \boldsymbol{x}_m \in \mathcal{M}, \Delta \boldsymbol{x}_c \in \mathcal{C}$ it holds that $d_m(\boldsymbol{x}_{m,1}, \mathbf{x}_{m,2}; \boldsymbol{\theta}) < d_m(\boldsymbol{x}_{c,1}, \mathbf{x}_{c,2}; \boldsymbol{\theta})$. It follows from Equation 3 that this is the case if the generative model learns to optimally separate the absolute differences of within-cluster instance pairs from across-cluster pairs.

In the generative **GDA** model Bensmail & Celeux (1996), the likelihoods of a pair of instances either being co-represented (i.e. belonging to the same sub-map), or not being co-represented (i.e. belonging to different sub-maps) are each modelled using a multivariate Gaussian:

$$p(\Delta \mathbf{x}|c = i; \mu_i, \Sigma_i) = (2\pi)^{-\frac{D}{2}} |\mathbf{\Sigma}_i|^{-\frac{1}{2}} \, e^{-\frac{1}{2}(\Delta \mathbf{x} - \boldsymbol{\mu}_i)^\intercal \mathbf{\Sigma}_i^{-1} (\Delta \mathbf{x} - \boldsymbol{\mu}_i)}, \tag{4}$$

where $i \in \{0, 1\}$. (μ_1, Σ_1) are the means and covariances of the APD distances of co-represented pairs, and (μ_0, Σ_0) those of not co-represented

pairs (see the two Gaussian contours in Figure 5 in the main text). These parameters can be easily estimated from the two given sets of co-represented and not co-represented object pairs, respectively, by calculating their means and covariances.

Finally, the trained GDA-model defined in equation (4) can be used to calculate distances between all pairs of objects in any testing data set. The data is projected under this metric using distance-preserving embedding. We have used multi-dimensional scaling for this purpose (Borg & Groenen, 2005). The result of this projection is a data set embedded such that Euclidean pairwise distances therein reflect the distances (3) in the original dataset.

We subsequently perform clustering of this resulting data, using a Dirichlet Process Gaussian Mixture Model (DP-GMM) (Rasmussen, 1999), since the number of clusters is unknown. Briefly, the DP-GMM model can be defined as follows:

$$\begin{aligned} \phi_k &\sim Beta(1, \alpha_1) \\ \mu_k &\sim Normal(0, \mathbf{I}) \\ \Sigma_k &\sim Wishart(D, \mathbf{I}) \\ \pi_k &\sim SBP(\phi) \\ x_t &\sim Normal(\mu_{z_i}, \Sigma_{z,i}^{-1}), \end{aligned} \tag{5}$$

where SBP stands for the stick-breaking process for generating mixture weights: $\pi_k = v_k \prod_{j=1}^{k-1}(1 - v_j)$. Data can be generated from this model by first choosing a cluster with probabilities specified by mixture weights: $z \sim Cat(\pi)$, and then drawing an observation from the parameters of that cluster $x \sim Normal(\mu_z, \Sigma_z)$.

Given the data, the parameters of this model can be inferred using variational inference (Blei et al., 2006). We have used the *bnpy* Python library for this purpose - see (Hughes & Sudderth, 2013) for further implementation details.

References

Baghshah, M. S., & Shouraki, S. B. (2010). Kernel-based metric learning for semi-supervised clustering. *Neurocomputing*, *73*, 1352–1361.

Bensmail, H., & Celeux, G. (1996). Regularized gaussian discriminant analysis through eigenvalue decomposition. *Journal of the American statistical Association*, *91*, 1743–1748.

Blei, D. M., Jordan, M. I. et al. (2006). Variational inference for dirichlet process mixtures. *Bayesian analysis*, *1*, 121–143.

Borg, I., & Groenen, P. J. (2005). *Modern multidimensional scaling: Theory and applications*. Springer Science & Business Media.

Breiman, L. (2001). Random forests. *Machine learning*, *45*, 5–32.

Chitta, R., Jin, R., Havens, T. C., & Jain, A. K. (2011). Approximate kernel k-means: Solution to large scale kernel clustering. In *Proceedings of the 17th ACM SIGKDD international conference on Knowledge discovery and data mining* (pp. 895–903). ACM.

Hirtle, S., & Jonides, J. (1985). Evidence of hierarchies in cognitive maps. *Memory & Cognition*, *13*, 208–217.

Hughes, M. C., & Sudderth, E. (2013). Memoized online variational inference for dirichlet process mixture models. In *Advances in Neural Information Processing Systems* (pp. 1133–1141).

Van der Maaten, L., & Hinton, G. (2008). Visualizing data using t-sne. *Journal of Machine Learning Research*, *9*, 85.

McNamara, T. P. (1986). Mental representations of spatial relations. *Cognitive psychology*, *18*, 87–121.

McNamara, T. P., Hardy, J. K., & Hirtle, S. C. (1989). Subjective hierarchies in spatial memory. *Journal of Experimental Psychology: Learning, Memory, and Cognition*, *15*, 211.

Ong, C. S., Williamson, R. C., & Smola, A. J. (2005). Learning the kernel with hyperkernels. In *Journal of Machine Learning Research* (pp. 1043–1071).

Rasmussen, C. E. (1999). The infinite gaussian mixture model. In *NIPS* (pp. 554–560). volume 12.

Xing, E. P., Jordan, M. I., Russell, S., & Ng, A. Y. (2002). Distance metric learning with application to clustering with side-information. In *Advances in neural information processing systems* (pp. 505–512).
